# Classification, substrate specificity and structural features of D-2-hydroxyacid dehydrogenases: 2HADH knowledgebase

**DOI:** 10.1186/s12862-018-1309-8

**Published:** 2018-12-22

**Authors:** Dorota Matelska, Ivan G. Shabalin, Jagoda Jabłońska, Marcin J. Domagalski, Jan Kutner, Krzysztof Ginalski, Wladek Minor

**Affiliations:** 1 Department of Molecular Physiology and Biological Physics, University of Virginia, 1340 Jefferson Park Avenue, Charlottesville, VA 22908 USA; 2 Laboratory of Bioinformatics and Systems Biology, Centre of New Technologies, University of Warsaw, Zwirki i Wigury 93, 02-089 Warsaw, Poland; 3 Center for Structural Genomics of Infectious Diseases (CSGID), Charlottesville, VA 22908 USA; 4 Laboratory for Structural and Biochemical Research, Biological and Chemical Research Centre, Department of Chemistry, University of Warsaw, Zwirki i Wigury 101, 02-089 Warsaw, Poland; 5 Department of Chemistry, University of Warsaw, Ludwika Pasteura 1, 02-093 Warsaw, Poland

**Keywords:** D-isomer specific 2-hydroxyacid dehydrogenases, Substrate specificity, Sequence-structure-function relationship, Substrate promiscuity, Molecular evolution

## Abstract

**Background:**

The family of D-isomer specific 2-hydroxyacid dehydrogenases (2HADHs) contains a wide range of oxidoreductases with various metabolic roles as well as biotechnological applications. Despite a vast amount of biochemical and structural data for various representatives of the family, the long and complex evolution and broad sequence diversity hinder functional annotations for uncharacterized members.

**Results:**

We report an in-depth phylogenetic analysis, followed by mapping of available biochemical and structural data on the reconstructed phylogenetic tree. The analysis suggests that some subfamilies comprising enzymes with similar yet broad substrate specificity profiles diverged early in the evolution of 2HADHs. Based on the phylogenetic tree, we present a revised classification of the family that comprises 22 subfamilies, including 13 new subfamilies not studied biochemically. We summarize characteristics of the nine biochemically studied subfamilies by aggregating all available sequence, biochemical, and structural data, providing comprehensive descriptions of the active site, cofactor-binding residues, and potential roles of specific structural regions in substrate recognition. In addition, we concisely present our analysis as an online 2HADH enzymes knowledgebase.

**Conclusions:**

The knowledgebase enables navigation over the 2HADHs classification, search through collected data, and functional predictions of uncharacterized 2HADHs. Future characterization of the new subfamilies may result in discoveries of enzymes with novel metabolic roles and with properties beneficial for biotechnological applications.

**Electronic supplementary material:**

The online version of this article (10.1186/s12862-018-1309-8) contains supplementary material, which is available to authorized users.

## Background

D-2-hydroxyacid dehydrogenases (2HADHs) constitute a widespread family of oxidoreductases, catalyzing the stereospecific, reversible reduction of 2-keto acids to the corresponding 2-hydroxy acids by the simultaneous oxidation of nicotinamide adenine dinucleotide (NAD^+^):

$$ \mathrm{R}-\mathrm{CO}-\mathrm{CO}\mathrm{OH}+\mathrm{NAD}\left(\mathrm{P}\right)\mathrm{H}+{\mathrm{H}}^{+}\leftrightharpoons \mathrm{R}-\mathrm{CH}\left(\mathrm{OH}\right)-\mathrm{CO}\mathrm{OH}+\mathrm{NAD}{\left(\mathrm{P}\right)}^{+}. $$

2HADHs can act either as reductases or dehydrogenases, use NADP(H) or NAD(H) as a cofactor, and possess varied substrate specificities. Due to their diversity of accepted substrates, the enzymes are implicated in different cellular processes, e.g., antibiotic resistance [1], photorespiration [2], or anaerobic glycolysis [3]. In humans, glyoxylate reductase (GRHPR) plays a critical role in the removal of the metabolic by-product glyoxylate from the liver [4]. Mutations in the *GRHPR* gene were found to cause primary hyperoxaluria type II, a rare disease characterized by endogenous overproduction of oxalate [4].

Most sequenced genomes encode multiple 2HADH paralogs. For example, the ɑ-proteobacterium *Sinorhizobium meliloti* has 16 paralogs, *Arabidopsis thaliana* has nine, *Escherichia coli* has five, and the human genome has four. Unfortunately, the exact biological function of the majority of these proteins is unknown because functional annotations of 2HADHs in protein databases rely on activities obtained for a small subset of selected substrates or on annotations available for the closest characterized homologs. Despite previous efforts [5–7], there is no consistent and comprehensive classification of 2HADHs into subfamilies. Moreover, no systematic studies show to what extent properties of studied members can be inter- or extrapolated, hindering assignment of biological processes and substrates. Thus, it is often difficult to predict the type of processes that uncharacterized 2HADH members are associated with. Better predictions and annotations would be particularly helpful for studies of medically relevant organisms, which often have several 2HADH enzymes with unclear functions. In addition, they will help to discover desired enzymes of potential biotechnological applications among a large number of environmental sequences collected from metagenomic samples.

Beyond their multiple cellular functions, 2HADHs have already been shown to possess a range of biotechnology applications. Enantiomerically pure 2-hydroxy acids are versatile building blocks for the synthesis of a variety of significant chiral compounds, which can be used as antimicrobial compounds [8], antitumour antibiotics [9], biodegradable polymers [9] or angiotensin-converting inhibitors [10]. As some 2HADHs can reduce a broad spectrum of 2-keto acids with high efficiency, they are used in systems for highly stereoselective production of selected chiral α-hydroxy carboxylic acids [11, 12]. Furthermore, formate dehydrogenase is used for efficient NADH regeneration in bioreduction systems [13], stimulation of certain metabolic pathways on a cellular level [14], and reduction of the atmospheric CO_2_ level [15]. Nevertheless, despite of the amount of biochemical, structural, and genomic data, finding or engineering stable and efficient enzymes for particular biotechnological processes have been difficult. Comprehensive classification of the family will help identification of highly efficient and thermodynamically stable enzymes for selected biotechnological processes, and better understanding of functional roles of different structural regions will guide rational design of such biocatalysts.

To better guide functional predictions, rational design, and new applications of these highly important enzymes, we analyzed biochemical and structural information available for 2HADH members in the light of their evolution. We systematically describe the active site, cofactor-binding residues, and potential roles of specific structural regions in substrate recognition for all the nine biochemically studied subfamilies. Furthermore, we provide a web-based knowledgebase to facilitate functional annotation of uncharacterized members and guide finding of enzymes with particular biochemical characteristics.

## Results

### A high-quality phylogenetic tree of the 2HADH family

**Fig. 1:**
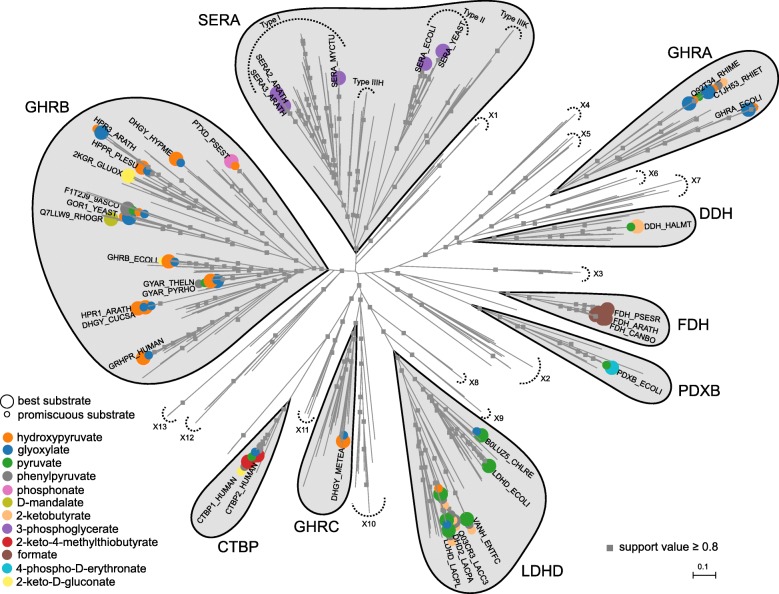
A maximum-likelihood tree of the 2HADHs from 111 representative organisms. The tree was computed with FastTree 2.1.7 [106] based on a high-quality, structure-based multiple sequence alignment and visualized with Archaeopteryx [108]. The separated subfamilies were defined based on high support values of the corresponding bifurcations and consistency between trees computed using different approaches. Proteins studied biochemically are marked with circles, which denote their substrates (large, most efficient in terms of *k*_cat_/*K*_M_; small, additional). SERA, 3-phosphoglycerate dehydrogenases; FDH, formate dehydrogenases; CTBP, C-terminal binding proteins; PDXB, 4-phosphoerythronate dehydrogenase; LDHD, D-lactate dehydrogenases; GHRA, glyoxylate/hydroxypyruvate reductases A; GHRB, glyoxylate/hydroxypyruvate reductases B; GHRC, glyoxylate/hydroxypyruvate reductases C; DDH, broad-substrate-specificity dehydrogenases; and X1-X13, subfamilies not studied biochemically. Nodes with local support values greater than 0.8 are denoted by grey squares. The tree in Newick format with branch support values can be found in Additional file 3: Data file S1

We calculated multiple phylogenetic trees in various ways (using neighbor-joining and maximum-likelihood approaches) and used nodes with high split support values (i.e., greater than 0.5) to assign sequences to subfamilies. Here, we define “subfamily” as a group of proteins that appear consistently as a clade in all the phylogenetic trees, which presumably share a similar function. Although low support values for the bifurcations close to the mid-point root indicate uncertainty of the path of the early evolution of the 2HADH family, the major subfamilies appear consistently as separate clades in the computed trees (Fig. 1). The 22 identified subfamilies include nine in which at least one member has been studied biochemically. Five of these subfamilies appeared in the previous classification [5]: 3-phosphoglycerate dehydrogenases (SERA), formate dehydrogenases (FDH), C-terminal binding proteins (CTBP), 4-phosphoerythronate dehydrogenase (PDXB), and D-lactate dehydrogenases (LDHD).

Due to little sequence similarity among distant 2HADH members (as noted earlier [16, 17]), noteworthy differences in subfamily classification may arise from the use of alternative methodologies for phylogenetic reconstruction. Notably, three subfamilies were classified into a single glyoxylate/hydroxypyruvate reductase (GHPR) cluster in the earlier neighbor-joining phylogenetic trees based on ClustalW sequence alignments [5, 6]. Here, these subfamilies are referred to as: glyoxylate/hydroxypyruvate reductases A (GHRA; including GhrA from *E. coli*, GHRA_ECOLI), glyoxylate/hydroxypyruvate reductases B (GHRB; including GhrB from *E. coli*, GHRB_ECOLI, and PtxD from *Pseudomonas stutzeri*, PTXD_PSEST) and broad-substrate-specificity dehydrogenases (DDH; including DDH from *Haloferax mediterranei*, DDH_HALMT). In all reconstructed trees, DDH and GHRA appear as closely related, yet separated, clades. Similarly, the polyphyletic origin of the GHRB subfamily and the clade encompassing GHRA and DDH subfamilies is supported in all reconstructed trees. Although in previous studies some GHRA and GHRB members showed similar substrate profiles and were classified as one group [5], in our analyses, they consistently appear as distantly related clades, separated early in the evolution of the 2HADH family (Fig. 1). Within GHRB, we also found a significant premise for a horizontal gene transfer from bacteria to plants, potentially occurred after early diversification of mesangiosperms [18] (elaborated in Additional file 1: Supplementary Results).

Besides GHRA, GHRB, and DDH, a fourth clade includes an enzyme previously shown to act as a hydroxypyruvate/glyoxylate reductase. HprA from the facultative methylotroph *Methylobacterium extorquens* (DHGY_METEA) plays a central role in carbon assimilation, as it converts hydroxypyruvate to glycerate in a critical step of the serine cycle [19]. The corresponding subfamily, which we name glyoxylate/hydroxypyruvate reductases C (GHRC), comprises bacteria from various phyla as well as a methanogenic archaeon, *Methanococcus maripaludis*, and has not been featured in previous classifications.

Along with the nine studied subfamilies, 13 additional clades not studied biochemically (X1-X13), including eight with representatives with a determined 3D structure (Additional file 2: Figure S1), could be defined with high support values (Additional file 3: Data file S1). Single long branches were left outside the classification; however, if more sequences were added, they could constitute additional clades.

### Substrate specificity of the 2HADH enzymes

To systematically describe the properties of the 2HADH subfamilies, we collected enzymatic parameters for the characterized representatives from the available literature (Additional file 4: Table S1). The collected data include 77 enzyme-substrate pairs with determined catalytic efficiency, defined as *k*_cat_/*K*_M_, based on which 14 compounds are ‘best’ substrates for at least one enzyme. Although 2HADHs were usually studied against a just a few substrates, most of them can be considered as promiscuous enzymes based on the collected data. The only exception constitutes FDHs, for which no substrates other than formate were determined so far; FDHs work through a different reaction mechanism, without typical stages of acid-base catalysis [20, 21]. Cumulatively, the 2HADH proteins are versatile catalysts in vitro—in total, they were shown to accept 33 compounds with either cofactor (Additional file 4: Table S1). The median *k*_cat_/*K*_M_ for the ‘best’ substrates is 1.45 × 10^5^ M^− 1^ s^− 1^ (Additional file 5: Figure S2), thus 2HADHs can be considered as moderately efficient catalysts, as compared to global trends for enzymes [22]. Interestingly, the two subfamilies most conserved regarding sequence and function, FDH and CTBP, comprise the least efficient catalysts, characterized by *k*_cat_/*K*_M_ of 10^2^–10^3^ M^− 1^ s^− 1^ and ~ 10^3^ M^− 1^ s^− 1^, respectively (Additional file 5: Figure S2). On the other hand, some of the most divergent subfamilies, GHRB and LDHD, encompass the most promiscuous and efficient enzymes.

In the studied in vitro conditions, most of the 2HADH subfamilies comprise members acting as reductases towards 2-keto acids, with a simultaneous oxidation of NADH or NADPH. Only three subfamilies contain representatives natively working as dehydrogenases, i.e., towards formate (FDH), 3-phosphoglycerate (SERA) and 4-phospho-D-erythronate (PDXB). In addition, two dehydrogenases were described in the highly heterogeneous GHRB cluster, i.e., phosphonate dehydrogenase from *P. stutzeri* [23] and D-mandelate dehydrogenase from *Rhodotorula graminis* [24]; also, the only characterized member of the GHRC subfamily was shown to possess glycerate dehydrogenase activity [19]. Unlike reductases, which have preferences for either NADH or NADPH, almost all wild-type dehydrogenases efficiently employ only NAD^+^ as a cofactor, which is expected given the typical redox state of a cell [20]. However, some formate dehydrogenases have been shown to possess dual cofactor specificity (i.e., working with NAD^+^ and NADP^+^) [19, 25].

In general, the data suggest that well-evolved enzyme-substrate interactions are rather rare among 2HADHs. Median affinity to the “best” (i.e., catalyzed with the highest efficiency) substrates, approximated as *K*_M_ (or *K*_1/2_ in case of non-Michaelis-Menten behavior), is lower than the average affinity for metabolic enzymes in general (*K*_M_ of 600 μM against 130 μM [22, 26], respectively). Only a few 2HADHs display higher affinity (compared to the global average) for their native substrates. Most of these are promiscuous 2HADH enzymes, with *E. coli* PdxB in the extremum (*K*_M_ of 2.9 μM, Additional file 5: Figure S2) [27]. As described for other enzyme families [26], a substrate considered as the physiological or most efficient in vitro in one subfamily is often secondary in other subfamilies (Fig. 1). In 2HADHs, glyoxylate, hydroxypyruvate, and pyruvate recurrently appear as accepted substrates in most subfamilies.

### Analysis of crystal structures

Besides kinetics, a wealth of structural data is also available for 2HADH enzymes. Out of the 22 defined subfamilies, 16 contain representative proteins with solved crystal structures (Additional file 2: Figure S1). Among the 121 2HADH structures available in the PDB, 40 were solved with both a cofactor and a ligand bound in the active site (Additional file 6: Table S2). Nevertheless, almost half of the structures have not been discussed in the literature (as indicated by the lack of a linked reference publication in the PDB). Several subfamilies (DDH, GHRC, X4, X6, X7, X9, X10, X12, and X13) have representative structures available in the PDB, but not a single one was presented in the scientific literature.

**Fig. 2:**
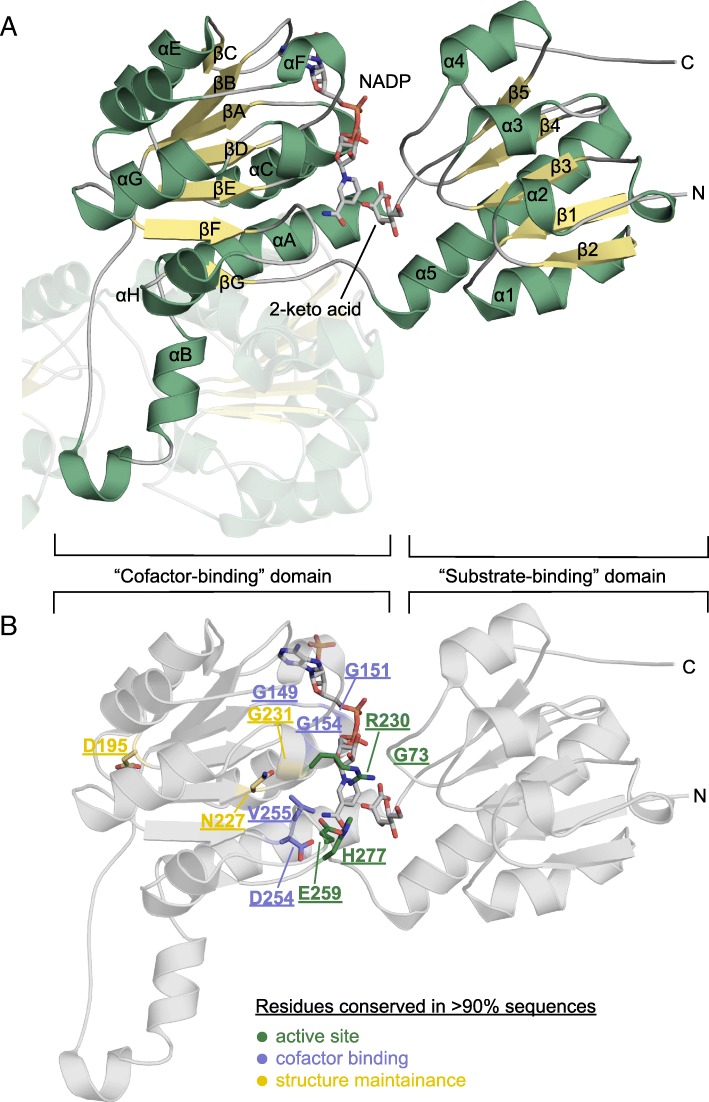
Crystal structure of a 2HADH from *Sinorhizobium meliloti* (PDB ID: 5v7n) complexed with a cofactor (NADP^+^) and a reaction product (2-keto-D-gluconic acid). Cofactor-binding and substrate-binding domains are indicated by brackets. **a**, Secondary structure elements are labeled; the other subunit of the dimer is translucent. **b**, Highly conserved residues (> 90% in all 2HADH sequences) are labeled

2HADHs usually act as homodimers, where each monomer is composed of two domains: a cofactor-binding domain with a classical NAD(P)-binding Rossmann fold, and a substrate-binding (or catalytic) domain with a modified (flavodoxin-like) Rossmann fold [28] (Fig. 2). The cofactor-binding domain is embedded in the substrate-binding domain and characterized by a more conserved sequence. The active site is located in the cleft formed between the two domains and is built mainly with residues from the cofactor-binding domain. According to solved crystal structures of apo and holo forms, 2HADHs can exist in either “open” and “closed” conformational states. Transition from the open to the closed conformation is essential for the formation of the enzyme active site and for catalysis [29]. Generally, crystal structures of 2HADHs without the cofactor bound display the open conformation, and holo forms display the closed conformation. However, there are a few exceptions to that general trend, which are likely caused by compounds present in the crystallization cocktails (e.g., sulfates) and different crystal environments [30]. The general consensus is that the 2HADH enzymes are in a dynamic equilibrium between the open and closed states and that the binding of cofactor shifts the equilibrium towards the closed state [29]. Because the substrate binds to residues from both domains, its binding is likely to contribute to shifting the equilibrium towards the closed state.

The structure-based alignment of representative sequences shows conservation of several residues (Additional file 7: Figure S3), suggesting a crucial role for these amino acids across the whole family. Some of them are well known to perform crucial functions, yet others were not previously discussed in the literature (e.g., Val72, Gly73, Asn227, Gly229, and Gly231, see below). The importance of some of these residues could only be determined by family-wide sequence comparison, as opposed to analyses of single structures, which only highlight important features of a particular enzyme. We divide the functions of highly conserved residues (> 90% conservation across all 2HADH sequences) into three categories—residues binding the cofactor, contributing to catalysis, and maintaining overall structure (Fig. 2). In the following analysis, the sequence numbering is according to a representative enzyme from the GHRB subfamily, Q92LZ4_RHIME (PDB ID: 5v7n).

#### Residues binding the cofactor

**Fig. 3:**
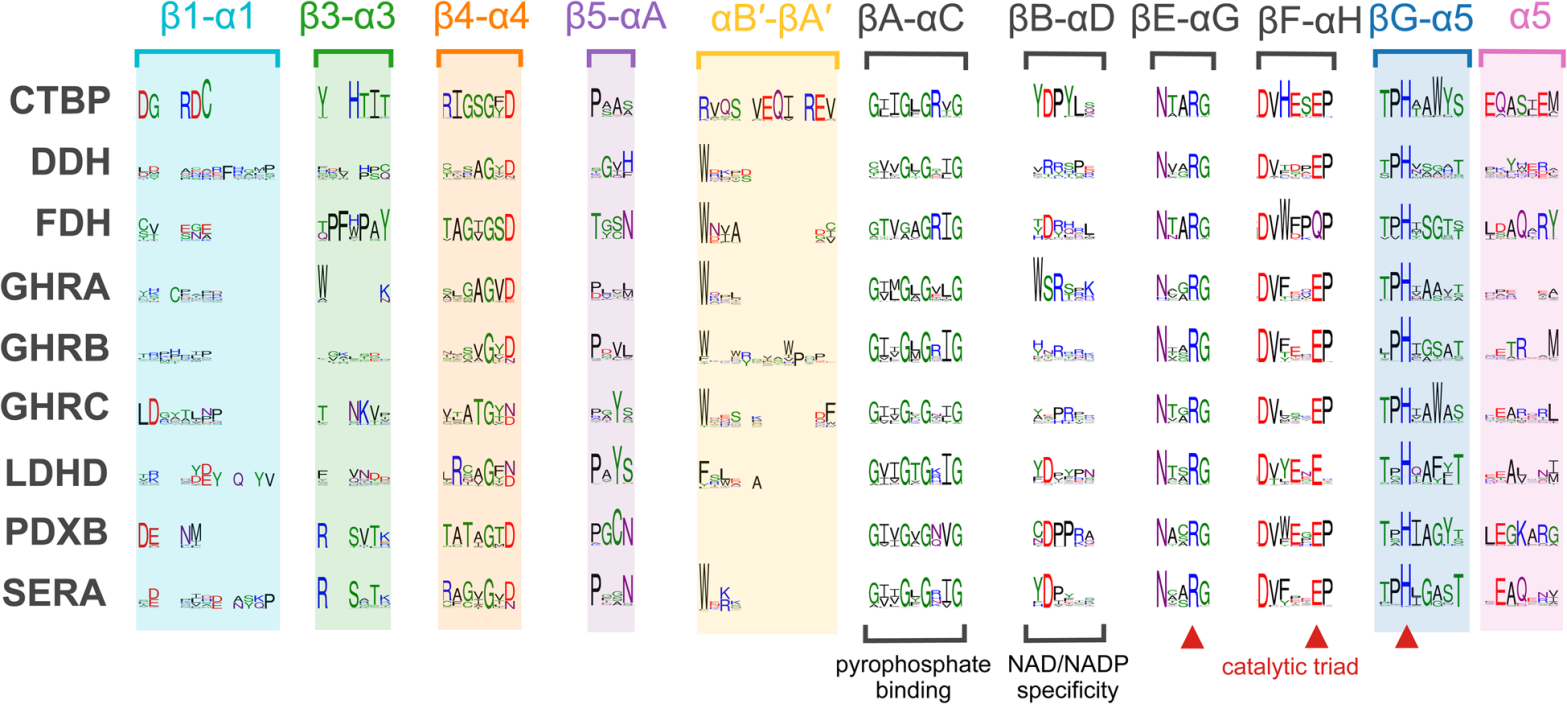
Sequence logos of selected regions critical for substrate and cofactor binding in the nine biochemically studied 2HADH subfamilies. The structure-based alignment was obtained for selected structures with PROMALS3D and used as a seed alignment for other 2HADH sequences from 111 representative organisms. The sequence logos were generated with WebLogo, showing columns for which in at least one subfamily at least 90% members possess an amino acid (i.e., with at most 10% gapped positions). Rectangles with colored backgrounds comprise loops implicated in substrate specificity. Catalytic triad residues are denoted by red triangles. Sequence logos of the full-length alignments are shown in Additional file 7: Figure S3

Among the residues crucial for cofactor-binding, a highly conserved pyrophosphate-binding GXXGXGXXG motif (residues 146–154 in Q92LZ4_RHIME) is common for Rossmann-fold dinucleotide-binding proteins. The motif is located in the region connecting the first strand of the β-sheet to the α-helix of the Rossmann fold (i.e., βA-αC). It contributes to the structural arrangement of the pyrophosphate bridge of the cofactor by assuring nearly optimal dihedral angles [31]. Although mutations of the glycine residues result in a drastic loss of enzymatic activity [32], neither of them are totally conserved among all 2HADH sequences (Fig. 3). It was shown that an Ala-to-Gly mutation improves protein thermal stability and decreases *K*_M_ towards NADH [33]. Two other conserved residues, aspartate (Asp254) and valine (Val255) contribute to binding of the pyridine ring of the cofactor [34, 35].

The specificity towards the cofactor [NAD(H) vs. NADP(H)] is effectively defined by the residues located in the βB-αD loop at the pocket that binds adenine and ribose moieties of the cofactor (Fig. 3). Five of the nine biochemically characterized subfamilies (CTBP, FDH, LDHD, PDXB, and SERA) have a highly conserved aspartate residue in this region, corresponding with a preference for NADH [36]. Many of GHRA enzymes have the characteristic motif [ST]R[ST]X[RK] in the same βB-αD loop – a conserved sequence fingerprint corresponding with specificity towards NADP(H). Other three biochemically characterized subfamilies do not have highly conserved residues in the region, suggesting varying cofactor specificity within these subfamilies.

#### Residues of the active site

The catalytic mechanism for the NAD^+^-dependent oxidation of 2-hydroxy acids (and the reverse reduction) is dependent on an internal acid-base catalyst, typically histidine [37, 38]. During the oxidation reaction, a hydride ion leaves the C2 atom of a substrate and attacks the C4 atom of the NAD^+^ pyridine moiety, and a proton moves from the hydroxy group of the substrate to the histidine of the active site [29]. Together with the histidine, two more residues within the active site—Arg and Glu/Asn—are thought to contribute directly to the reaction and are referred to as the “catalytic triad” [39]. The highly conserved arginine stabilizes and polarizes the bound substrate, whereas the glutamate (or asparagine in formate dehydrogenases) stabilizes the protonated form of the catalytic histidine.

**Fig. 4:**
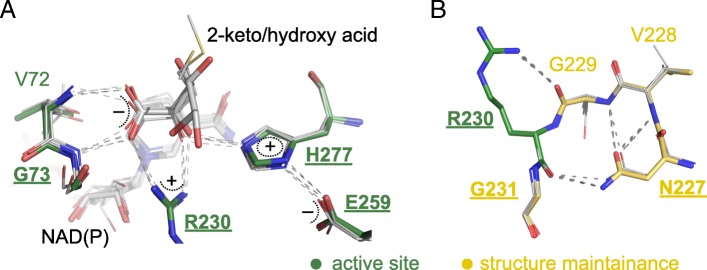
Active site of canonical 2HADHs: (**a**), active site residues, reaction substrates/products (2-keto acids/2-hydroxy acids), and cofactors [NADP(H) or NAD (H))]; (**b**), structural support of the active site arginine. Shown are selected residues of five ternary complexes: *S. meliloti* GHRB with 2-keto-D-gluconic acid and NADP^+^ (PDB ID: 5v7n, shown in wider sticks), human GRHPR with 2,3-dihydroxypropanoic acid and NADP^+^ (PDB ID: 2gcg), human CTBP1 with 4-methylthio-2-oxobutyric acid and NAD^+^ (PDB ID: 4lce), human CTBP2 and 4-methylthio-2-oxobutyric acid and NAD^+^ (PDB ID: 4lcj), and *A. aeolicus* subfamily X9 member with lactic acid and NAD^+^ (PDB ID: 3 kb6). Oxygen and nitrogen atoms are shown in blue and red, respectively, with carbon atoms in green (for PDB ID: 5v7n) or gray (in other structures). Hydrogen bonds between protein residues and product are shown with gray dashed lines. Residues are labeled according to the sequence of PDB ID: 5v7n. Labels of highly conserved residues (i.e., present in > 90% of 2HADH sequences) are shown in bold and underlined

The mode of the substrate binding was subject to long discussions over several years; at least three modes of 2-keto/2-hydroxy acid binding were proposed [29, 34]. As our analysis of crystal structures shows, despite 40 structures being solved with both a cofactor and a ligand bound in the active site, only eight of them represent a true ternary complex—a complex with a reduced cofactor and a reduced substrate, or with an oxidized cofactor and an oxidized substrate (Additional file 6: Table S2). The remaining triple complex structures have an inhibitor, a substrate analog, or a solute bound in the active site and do not provide a complete model of substrate binding (except for FDH, whose substrate is not a 2-hydroxy acid), as discussed earlier [29]. In 2006, the first crystal structure of a true ternary complex of a 2HADH (human GHRB, GRHPR_HUMAN, PDB ID: 2gcg) was published [38], demonstrating the interactions between the substrate and catalytic residues within the active site and confirming one of the earlier proposals for the mode of substrate binding (Fig.4). Later, the same mode of substrate binding was observed in all other 2HADH true ternary complexes with clearly observed electron densities for the ligands: human CTBPs (CTBP1_HUMAN and CTBP2_HUMAN) in complex with NAD^+^ and 4-methylthio-2-oxobutyric acid (PDB ID: 4lce and 4lcj) [40], GHRB from *Rhizobium meliloti* (Q92LZ4_RHIME) in complex with NADP^+^ and 2-keto-D-gluconic acid (PDB ID: 5v7n), and an enzyme from *Aquifex aeolicus* that belongs to the X9 subfamily (O66939_AQUAE) in complex with a cofactor and unknown ligand, interpreted as a complex with NADH and lactate (PDB ID: 3 kb6) [41] (Fig. 4). As seen in Fig. 4, one of the ligands (4-methylthio-2-oxobutyric acid bound to CtBP1 in complex with NAD^+^; PDB ID: 4lce) is modeled in a slightly unusual orientation, with the carboxyl group rotated around the C1-C2 bond. This ligand corresponds to the unusual C2′-endo conformation of the ribose moiety of the cofactor seen in the same figure. However, both the ligand and the cofactor show incomplete occupancy and poor electron density in the crystal structure and should be interpreted with caution. To address this issue, we downloaded the structural model and structure factors of 4lce that were deposited in the PDB and re-refined this structure using recently published guidelines [42]. Re-refinement revealed that the sugar moiety is likely in the C3′-endo conformation (as in all other 2HADH structures) and that the orientation of the keto-acid is largely consistent with other structures.

These structures show the mode of 2-keto/2-hydroxy acid binding by 2HADH that involves four highly conserved residues (Gly73, Arg230, Glu259, and His277, conserved in more than 90% of all 2HADH sequences) and a variable residue that bind the substrate via a main-chain amide (Val72). Two consecutive main chain amines from the β4-α4 loop (Fig. 3, Fig. 4) form hydrogen bonds to the carboxylate atoms of the substrate, positioning the carboxyl group and thus orienting the substrate relative to the cofactor. The first amide comes from Val72, which is often replaced with another small residue such as alanine, serine, or threonine (Additional file 7: Figure S3). The second amide comes from a highly conserved glycine (Gly73). The only exception from this general pattern is subfamily X13, which has asparagine and leucine residues in these consecutive positions, respectively. Arg230 is the most conserved residue in the active site because only arginine provides the positively charged guanidinium group that can bind the substrate via two atoms and thus properly orient the substrate in addition to stabilizing its charge. The guanidinium group binds both the reducible/oxidizable keto/hydroxyl oxygen, presumably leading to its polarization, and the single oxygen of the substrate carboxylate, thus contributing to the orientation of the substrate [38]. His277 is involved both in substrate binding and a “proton shuttle” system between the histidine and the carboxylic acid residue Glu259. Notably, the four residues responsible for direct binding of the core of the substrate belong to both the catalytic (Val72 and Gly73) and the cofactor-binding domains (Arg230 and His277), thus making the interdomain cleft closure a necessary prerequisite for catalysis. The roles of the active site residues in the catalysis are supported by multiple mutagenesis studies [21, 43–46].

The residues Arg230, Glu259, and His277, often referred to as the “catalytic triad” [39] are conserved in almost all 2HADHs (Fig. 3). However, there are single cases of substitutions in these positions. In most FDHs, glutamate is substituted with glutamine, which broadens the optimal pH range for substrate binding [46]. Histidine, which is thought to exchange a proton in the redox reaction, is substituted by lysine in SERA type IIIK, and by tryptophan in X4 (PDB ID: 4njo and 1qp8, respectively; Additional file 8: Figure S4). Despite the histidine substitution for lysine and the absence of glutamate, a SERA type IIIK enzyme was shown to be catalytically active, presumably because lysine is also capable of maintaining two protonated states of the side chain [47]. However, the indole nitrogen in tryptophan is never protonated under physiological conditions (p*K*_a_ = − 2.4) and it cannot lose the proton; thus, it cannot serve as a catalytic residue that would provide a proton for the catalysis. The molecular function of the members of X4 with tryptophan instead of histidine should still be studied experimentally (see section: New uncharacterized subfamilies).

#### Residues maintaining the structure

Among the three conserved residues with structural function, asparagine (Asn227) and glycine (Gly231), located in the βE-αG loop, are responsible for positioning and conformational stabilization of the catalytic arginine Arg230 (Fig. 4). Gly231, which follows Arg230, gives the conformational flexibility to the protein main chain that is necessary to position the arginine side chain in the proper orientation. Asn227, separated by two residues from the arginine, locks the arginine main chain by forming hydrogen bonds to its main chain oxygen and to the main chain nitrogen of Gly229. Asn227 is highly conserved in 2HADHs, because asparagine side chain has the capacity of forming the two hydrogen bonds with the main chain atoms (one atom accepts hydrogen, and the other is donor, Fig. 4). This highly specific conformation of the polypeptide chain requires extra conformational flexibility of the main chain around the residue preceding Arg230, which is provided by glycine (Gly229). Interestingly, Gly229 is not highly conserved in 2HADHs and is often replaced by a residue with a small side chain (Ala, Ser). However, these residues are always found to be Ramachandran plot outliers in all known crystal structures (PDB IDs: 5tx7, 5dt9, 3oet, 2o4c, and others).

The third residue, located downstream of the crossover helix αE of the cofactor-binding domain, usually aspartate (93% of the sequences; Asp195), is substituted to arginine in 3% of the sequences. This residue forms hydrogen bonds to residues in adjacent loops, probably contributing to the maintenance of the fold.

#### Residues contributing to substrate specificity

Based on the collected data, an enzyme with narrow substrate specificity in the 2HADH family is exceptional, and different subfamilies, separated early in evolution, often exhibit similar substrate profiles (Fig. 1, Additional file 4: Table S1). Therefore, determination of positions crucial for substrate specificity (which we term “specificity determining positions”) that are general for the entire family is a particularly difficult or, maybe even impossible, task. Previous analyses of solved crystal structures and sequence alignments suggested that specific residues govern substrate discrimination within single subfamilies [29, 35, 38, 39]. In addition, some attempts were made to change substrate specificity of single enzymes by introducing point mutations in the proximity of the active site [48]. However, the mutagenesis data is scarce and hypotheses about molecular features governing substrate specificity among 2HADHs are largely based on crystal structures of ternary complexes solved with inactive substrate analogs or products.

Substrate specificity stems largely from the acquisition of unique loop regions and the adaptation of the physico-chemical nature of the substrate-binding pocket. Our analysis of available crystal structures shows that residues that can contact a variable substituent at the C2 carbon atom are found—depending on the structure and substrate—in up to eight regions of the sequence (Fig. 3, Additional file 8: Figure S4). Four of them are supplied by the substrate-binding domain (loops β1-α1, β3-α3, β4-α4, and helix α5). Two regions are located within loops connecting the two domains (loops β5-αA and βG-α5). Selectivity in 2HADH also appears to be dependent on the interactions within oligomeric assemblies: in many complexes, some residues in the substrate pocket, usually aromatic or acidic, are supplied by another subunit of a dimer (i.e., αB′-βA′). Furthermore, in PDXB, a dimerization domain specific to PDXB can supplement the pocket with a substrate-binding arginine residue (PDB ID: 2o4c, “PDXB_dim” in Additional file 7: Figure S3).

Due to local structural changes of the enzymes, the broad range of physicochemical properties of their substrates, and the location of the catalytic pocket at the interface of two domains, 2HADH subfamilies developed various modes of substrate discrimination (Additional file 8: Figure S4). Below, we provide the first attempt to systematically characterize the contributions of specific regions of 2HADH structures to the substrate specificity of subfamilies, based on available crystal structures. However, as noted before for other enzyme families, substrate specificity may go beyond the physicochemical and steric characteristics of the active site, i.e., it may depend on global protein dynamics, the transition from the ‘close’ to ‘open’ conformation, and the mechanism of substrate entrance/exit [49, 50].

### Descriptions of the subfamilies

**Fig. 5:**
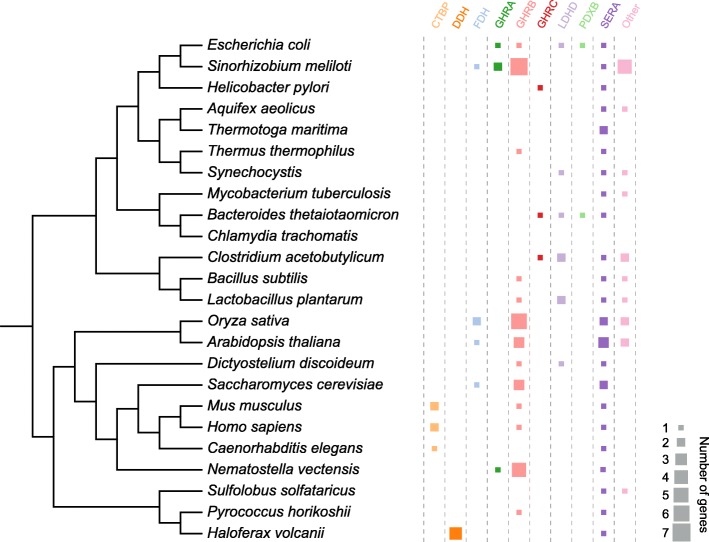
Abundance of the nine biochemically studied 2HADH subfamilies in selected model organisms. The size of each square corresponds to the number of proteins belonging to a given subfamily encoded in the given organism. The tree topology was obtained from iTOL [112], and proteomes were downloaded from KEGG [113] (Additional file 9: Data file S2)

The properties of the nine biochemically studied subfamilies (Fig. 1) are summarized in Table 1. Figure 3 shows the sequence logos of their regions critical for substrate and cofactor binding. Figure 5 shows their abundance in the genomes of model organisms.

**Table 1:** Descriptions of the nine biochemically studied 2HADH subfamilies. Numbers in parentheses in the column “Accepted substrates” denote the number of enzymes shown to accept a given substrate, if more than one (see Additional file 4: Table S1 for details)

Subfamily	Name	Description	Taxonomy	Postulated biological functions	Accepted substrates	Cofactors
CTBP	C-terminal binding proteins	Human CtBP1 reduces a number of substrates with a relatively low activity, using NADH as a cofactor [50]. It shows the best catalytic efficiency with 2-keto-4-methylthiobutyrate, an intermediate of the methionine salvage pathway [50]. The saturation curve shows biphasic behavior, with marked substrate inhibition at elevated concentrations [50]. Physiological substrates for CTBP proteins are not known.	Eukaryotes (vertebrates, arthropods)	Transcriptional corepressors targeting many transcriptional regulators [51] and playing critical roles during development of both invertebrates and vertebrates [52]. They have intrinsic dehydrogenase activity and the NAD^+^-dependent conformational change is thought to be essential to their co-repression activity [53, 54]. Two copies (CTBP1_HUMAN, CTBP2_HUMAN) are encoded in the human genome. *A. thaliana* homolog (CTBP_ARATH, C-terminal binding protein AN), which is a sister clade to the CTBP family, differs substantially in sequence, lacks the catalytic residues and seems not to regulate transcription [55], therefore was excluded from the family.	2-keto-4-methylthiobutyrate (2), 3-phosphohydroxypyruvate, 2-keto-D-gluconate, 2-ketovalerate, pyruvate, 2-ketoisocaproate, 2-ketoglutarate, phenylpyruvate, glyoxylate, 2-ketocaproate, oxaloacetate	CTBP1_HUMAN functions equally effective with NADH and NAD^+^ [53, 54].
DDH	2-ketocarboxylic reductases with broad substrate specificity	ddh from *Haloferax mediterranei* catalyzes reduction of α-ketocarboxylic acids showing marked preference for those having an unbranched chain of 4–5 carbon atoms, such as 2-ketoisoleucine [56].	Eukaryotes (fungi, protists), archaea and bacteria (cyanobacteria, actinobacteria)	Function unknown. Four copies encoded in the genome of a halophilic mesophile, *Haloferax volcanii*.	pyruvate, 2-ketoisocaproate, 2-ketobutyrate, 2-keto-3-methylvalerate	DDH_HALMT prefers NADPH over NADH [56].
FDH	formate dehydroganases	A highly conserved group of enzymes, mostly specific to both formate and NAD^+^. Mechanism of the catalyzed reaction differs from that observed in other related dehydrogenases – it is specified by a direct transfer of hydride ion from the substrate onto the C4-atom of the nicotinamide moiety of NAD^+^ without stages of acid-base catalysis [21].	Eukaryotes (fungi, plants) and bacteria (Firmicutes, proteobacteria)	FDHs are involved in methanol utilization in all methylotrophic microorganisms (yeast and bacteria) [57] and in stress response in higher plants [58].	formate (5)	Majority FDHs are specific to NAD^+^ [57]. Some possess dual cofactor specificity, with NADP^+^ preferred over NAD^+^, e.g. G8NVB5_GRAMM [59] and B5A8W5_9BURK [25].
GHRA	glyoxylate/hydroxypyruvate reductases A	Bacterial (mostly) group of enzymes, studied biochemically in *E. coli* and *R. etli*. They show similar substrate specificity profiles, accepting glyoxylate, hydroxypyruvate, but not pyruvate, 2-ketoglutarate and 2-keto-D-gluconate [5, 60]. In addition, *R. etli* GxrA reduces phenylpyruvate and 2-ketobutyrate [5].	Bacteria (proteobacteria) and eukaryotes (arthropods, e.g., *Nematostella vectensis*)	Reduction of glyoxylate [60]. *E. coli* YcdW could be replaced by YiaE belonging to the GHRB subfamily [60].	hydroxypyruvate (3), glyoxylate (3), hydroxyphenylpyruvate, 2-ketobutyrate, pyruvate, phenylpyruvate	Majority sequences have the NADPH-binding motif. GHRA_ECOLI prefers NADPH over NADH [60], Q92T34_RHIME works only with NADPH [64], while C1JH53_RHIET only with NADH [5].
GHRB	glyoxylate/hydroxypyruvate reductases B	Heterogeneous and widely spread group of enzymes. They usually work most efficiently with glyoxylate and hydroxypyruvate, but not pyruvate (GRHPR_HUMAN, GHRB_ECOLI); however, some are more specific towards hydroxyphenylpyruvate (HPPR_PLESU). They group together with PTXD_PSEST, which oxidizes phosphonate, and D-mandalate dehydrogenase (Q9LLW9_RHOGR).	Eukaryotes, bacteria and archaea	In mammals, glyoxylate reductase, expressed primarily in kidney and liver, is involved in the serine degradation pathway [61]. GRHPR_HUMAN converts hydroxypyruvate to D-glycerate and glyoxylate to glycolate and mutations in the gene causes primary hyperoxaluria type II [4]. Hydroxyphenylpyruvate reductase in *Coleus blumei* (HPPR_PLESU), is involved in the rosmarinic acid biosynthesis [62], and hydroxypyruvate reductases in *A. thalian*a (HPR1_ARATH, HPR2_ARATH, HPR3_ARATH) in photorespiratory metabolism. In methylotrophic organisms, hydroxypyruvate reductase (DHGY_HYPME) plays a central role in carbon assimilation, converting hydroxypyruvate to glycerate as a key step in the serine cycle [63].	hydroxypyruvate (13), glyoxylate (12), phenylpyruvate (3), pyruvate (2), 4-hydroxyphenylpyruvate (2), hydroxyphenylpyruvate, oxaloacetate, 2-keto-D-gluconate, 2-hydroxyisocaproate, D-mandalate, 2-keto-L-gulonate, phenylglyoxylate, phosphonate, 3,4-dihydroxyphenylpyruvate, benzylformate, 2-keto-D-gluconic acid	Usually possess better affinity to NADPH than NADH (GRHPR_HUMAN [38], HPPR_PLESU [62], GHRB_ECOLI [63]), but some enzymes work better with NADH (HPR1_ARATH [68]).
GHRC	glyoxylate/hydroxypyruvate reductases C	An enzyme from a methylotroph *M. extorquens* was shown to reduce hydroxypyruvate and glyoxylate, and catalyze reverse reaction with glycerate but not glycolate [19].	Bacteria and archaea	It plays a central role in assimilation of carbon in methylotrophic organisms as it converts hydroxypyruvate to glycerate as a key step in the serine cycle, may also play an important role in C2 reactions by interconverting glyoxylate and glycolate [19].	hydroxypyruvate, glyoxylate, D-glycerate	DHGY_METEA is active with both NADH and NADPH [19].
LDHD	D-lactate dehydrogenases	According to the phylogenetic analysis, there are two subgroups within this clade: a Bacilli-specific clade and a clade comprising other bacteria and eukaryotes. Originally annotated as D-lactate dehydrogenases, work with a broad range of small substrates, but usually best with pyruvate, using NADH as a cofactor. However, 2-ketoisocaproate was shown to be the best substrate for the enzyme from *L. casei* [64]. *E. coli* LDHD was shown to be inhibited in situ by substrate in high concentrations [65]. VanH from *Enterococcus faecium* was shown to work best with pyruvate and 2-ketobutyrate [66], whereas relatively diverged *Chlamydomonas reinhardtii* D-LDH reduces pyruvate in chloroplasts and works as a tetramer [67].	Bacteria and lower eukaryotes (protists, fungi, green alga)	The Bacilli enzymes are postulated to reduce pyruvate, the final product of glycolysis, to lactate [68]. VanH from *E. faecium* is involved in vancomycin resistance [66]. *Chlamydomonas reinhardtii* D-LDH reduces pyruvate in fermentation pathways in chloroplasts [67].	pyruvate (8), 2-ketobutyrate (7), phenylpyruvate (7), 2-ketovalerate (4), 2-ketoisocaproate (4), 2-ketocaproate (4), lactate (3), 2-ketoisovalerate (3), hydroxypyruvate (2), glyoxylate (2), 2-keto-3-methylbutyrate, 2-keto-4-methylmercaptobutyrate, mercaptopyruvate, 2-ketooctanoate, 2-oobutanoate, 4-hydroxyphenylpyruvate, oxaloacetate, 2-ketovalerate, 2-ketohexanoate, bromopyruvate, 2-keto-3-methylvalerate	LDHD enzymes utilizes NADH as a cofactor [65, 67, 68].
PDXB	erythronate-4-phosphate dehydrogenases	*E. coli* PdxB oxidizes 4-phospho-D-erythronate to 2-keto-3-hydroxy-4-phosphobutanoate [69] and uses various 2-keto acids as co-substrates [27].	Bacteria (ɣ-proteobacteria and bacteroidetes)	In *E. coli*, PdxB catalyzes the second step in the biosynthesis of pyridoxal phosphate (active form of vitamin B6) [69].	α-ketoglutarate, 4-phospho-D-erythronate, pyruvate, oxaloacetate	PDXB_ECOLI utilizes NADH/NAD^+^ as a cofactor [69].
SERA	3-phosphoglycerate dehydrogenases	PGDHs can be divided into four distinct groups [70]. They convert D-3-phosphoglycerate to hydroxypyruvic acid phosphate. *E. coli* SerA is strongly inhibited by L-serine, the end product of the pathway, which binds to the ACT domain and allosterically regulates velocity of the catalyzed reaction [71]. Unlike *Mycobacterium tuberculosis* and rat SerA enzymes, *E. coli* SerA can also utilize α-ketoglutarate as a substrate, yet with considerably lower affinity than 3-phosphoglycerate [70].	Eukaryotes, bacteria and archaea	They catalyze the first committed step in the phosphorylated pathway of L-serine biosynthesis by converting D-3-phosphoglycerate to hydroxypyruvic acid phosphate [72].	3-phosphoglycerate (6), 3-sulfopyruvate, sulfolactate, 2-ketoglutarate	SERA enzymes utilize NAD^+^ as a cofactor [72].

#### CTBP

C-terminal binding proteins (CtBPs) can be found in vertebrates (e.g., rats and humans) and arthropods (e.g., *Drosophila melanogaster* [51]), yet members studied enzymatically include only two human paralogs. The animal CtBPs localize to both the nucleus and the cytoplasm, and much effort has been made to study transrepression pathways in which they may be involved [52].

CtBPs were first identified as transcriptional corepressors targeting many transcriptional regulators [53] and playing critical roles during development of both invertebrates and vertebrates [52]. Although the precise mechanism of the corepressor activity is still under investigation, it is known that CtBPs recognize the consensus PXDLS motif in DNA-binding and other transcription-related proteins [54, 55]. Later studies confirmed that they also possess dehydrogenase activity, and the NAD^+^-dependent conformational change is thought to be essential to their corepression activity [56, 57]. Human CtBP1 (CTBP1_HUMAN) reduces a number of substrates, including glyoxylate and pyruvate, with relatively low activity, using NADH as a cofactor [58] (βB-αD loop, Fig. 3). Human CtBP1 shows the highest catalytic efficiency with 4-methylthio-2-oxobutyric acid (MTOB), an intermediate of the methionine salvage pathway [58]. The saturation curve shows biphasic behavior, with marked substrate inhibition at elevated concentrations [58]. Nevertheless, the most relevant physiological substrates for CtBPs and their role in the corepressor function remain unclear.

The homolog from *A. thaliana* (CTBP_ARATH, C-terminal binding protein AN) differs substantially from the animal CtBPs in sequence, lacks the catalytic residues, and does not seem to regulate transcription [59]. For this reason, the plant homologs were not included in the CTBP subfamily and considered as its sister clade.

Based on the available crystal structures solved in complex with a cofactor and MTOB, it was observed that the sulfur atom of MTOB forms a sulfur–π interaction with tryptophan from the βG-α5 loop (Trp318 in PDB ID: 4lce, Additional file 8: Figure S4). This interaction is thought to confer specificity towards MTOB [40]. Other bulky residues, such as a conserved histidine and tyrosine from the βG-α5 loop, form the tight binding pocket and appear to constrain the size of substrates accepted.

In the case of CtBP1, the tetramer assembly is preceded by a dimeric intermediate, in which the tryptophan (Trp318) from the βG-α5 loop functions as a switch for effective dimerization following NAD^+^ binding (Additional file 8: Figure S4, PDB ID: 4lce) [60, 61]. Mutation of this residue to phenylalanine reduced dimerization and completely abolished tetramerization, what suggested that NAD(H)-dependent dimerization occurs with Trp318 required to effectively induce the strand switch, bringing the dimer pairs into a spatial context permissive for homotetramerization [61].

#### DDH

This subfamily is named after the only biochemically studied representative, D-2-hydroxyacid dehydrogenase (Ddh) from *Haloferax mediterranei* (DDH_HALMT), and comprises proteins spread over the taxonomic tree, including eukaryotes (e.g., fungi and protists), archaea and bacteria (cyanobacteria and actinobacteria). Ddh from *H. mediterranei* catalyzes the reduction of a broad range of 2-ketocarboxylic acids, with a preference for those having an unbranched chain of 4–5 carbon atoms, such as 2-ketoisoleucine [62]. It exhibits dual cofactor specificity, yet shows better catalytic efficiency with NADPH [62]. The sequence conservation within the βB-αD loop does not display the respective aspartate residue defining the specificity towards NAD(H) (Fig. 3), suggesting that most enzymes within the DDH subfamily would display preference towards NADPH. Although some archaeal genomes (e.g., the halophilic mesophile *Haloferax volcanii*, Fig. 5) encode as many as four DDH representatives, their function is not known.

Recently, three crystal structures of DDH_HALMT were solved in complex with combinations of NAD^+^, NADP^+^, NADPH, 2-ketohexanoic acid, and 2-hydroxyhexanoic acid (PDB IDs: 5mha, 5mh5, 5mh6). Although the DDH subfamily displays high sequence variability, some common features can be distinguished based on the sequence alignment with other 2HADH subfamilies (Fig. 3). For example, the β1-α1 loop harbors a considerably long insertion, which folds into a tightly packed 3/10-helix in the crystal structures. Furthermore, a highly conserved tryptophan within the αB-A loop from the other subunit (Trp122 in DDH_HALMT), which is thought to preclude larger substrates from binding to members of the GHRA, GHRB, and SERA subfamilies, is located far away from the active site (Additional file 8: Figure S4). In addition, small residues within the β5-αA loop would allow accommodation of large and hydrophobic substrates, whereas large residues facing the active site from the 3/10-helix (such as Arg14 in DDH_HALMT) could possibly prevent the binding of branched substituents (Additional file 8: Figure S4).

#### FDH

Formate dehydrogenases (FDHs) represent a highly conserved subfamily of enzymes, characterized by a scattered taxonomic distribution. They are present in various bacteria (i.e., Firmicutes and proteobacteria) and eukaryotes (plants, yeasts, and fungi), and catalyze the NAD^+^-dependent oxidation of formate to carbon dioxide. The aspartate residue that defines the specificity for NAD^+^ is conserved within the subfamily (βB-αD loop, Fig. 3). The formate oxidation is the simplest reaction catalyzed by 2HADHs, as it lacks the proton release step. It is specified by a direct transfer of hydride ion from the substrate onto the C4 atom of the nicotinamide moiety of NAD^+^. Since formate is not a 2-hydroxy acid, the mode of substrate binding in FDH differs from other 2HADHs [21]. FDHs constitute the most studied 2HADH subfamily, and their characteristics have been reviewed extensively [33, 63].

FDHs play a pivotal role in methanol utilization in methylotrophic microorganisms (yeast and bacteria), supplying them with energy and reducing equivalents [21]. As opposed to microbiological FDHs, which function in cytoplasm, plant FDHs localize to the mitochondria and are key players in the cell stress response caused by both exogenic and endogenic factors [33].

The vast majority of FDHs studied so far accept only formate as a substrate and NAD^+^ as a cofactor, though some were found to possess double cofactor specificity [25, 64]. A number of enzymes have also been shown to oxidize esters and thioesters of formic acids [63]; however, the physiological significance of the additional substrates has not been confirmed.

Interestingly, although FDHs are considered highly specific enzymes, they possess relatively low affinity to formate, characterized by a *K*_M_ of 3–10 mM [21]. Affinities to the cofactor are usually 1–2 orders of magnitude higher, with most *K*_M_ values ranging from 10 to 100 μM [27, 65, 66]. Similarly, catalytic efficiencies are relatively small, yet higher in bacterial FDHs than methylotrophic yeast FDHs. Improvement of the catalytic parameters of FDHs by genetic engineering is an important issue, as FDH enzymes are widely used for NADH regeneration in enzymatic syntheses of optically active compounds [21].

A wealth of structural data [21, 30, 35] and computational studies [67, 68] is available for the FDH subfamily, making it one of the most studied 2HADH subfamilies. As reflected by the high sequence similarity among its members (Fig. 1), their active site environment is almost invariable. A common feature of all FDHs is an extended β3-α3 loop harboring the PF[HW] P sequence motif, which appears to significantly reduce the size of the active site. The invariant prolines maintain the aromatic residues in stereochemically constrained positions, further supported by stacking of their aromatic rings (Additional file 8: Figure S4). In consequence, the active site remains rigid and tightly packed, perfectly tailored for accommodation of small substrates.

#### GHRA

In previous classifications [5, 6], this subfamily (represented by *Rhizobium etli* GxrA and *E. coli* GhrA) was classified jointly with GHRB. However, in our evolutionary trees, it consistently appeared as polyphyletic with GHRB and clustered closely with DDH. Also, even in the phylogenetic trees underlying the previous classification, GHRA emerged and separated early from GHRB [5, 6]. This subfamily comprises mostly bacterial enzymes (except for one protein from *Nematostella vectensis*, closely related to β-proteobacterial enzymes), of which three (from *E. coli*, *R. etli*, and *S. meliloti*) have been characterized biochemically [5, 69]. Unlike most GHRB members, which have hydroxypyruvate as a preferred substrate, they have been shown to work most efficiently towards glyoxylate. Their secondary substrates include hydroxypyruvate, phenylpyruvate and pyruvate, but not 2-keto-D-gluconate, which is a substrate for several GHRB members. In our recent study [70], we highlight differences between the GHRA and GHRB clades by structural and enzymatic characterization of two members from *S. meliloti* 1021.

Enzymes that belong to the GHRA clade exhibit high sequence similarity. They share a conserved sequence fingerprint for specificity towards NADPH at the pocket shown to bind adenine and ribose moieties of the cofactor ([ST]R[ST]X[RK] in the βB-αD loop, Fig. 3) [71]. In vitro, *E. coli* and *S. meliloti* representatives were indeed shown to be selective for NADPH over NADH. However, *R. etli* GxrA was reported to work only with NADH [5], which seems dubious, because the sequence of *R. etli* GxrA has the fingerprint of specificity for NADPH. The physiological function of the enzymes remains to be discovered. The *E. coli* GhrA was proposed to contribute to glyoxylate reduction in the cell, yet in a dispensable manner [69].

Crystal structures of the GHRA homologs bound with substrate analogs revealed a large hydrophobic active site with a conserved tryptophan from the β3-α3 loop (Trp53 in *R. etli*, *S. meliloti* or *Xanthobacter autotrophicus* GhrA, PDB IDs: 5tsd, 4z0p or 5vg6, respectively) interacting with C2 atom substituents. The tryptophan is unique to the GHRA subfamily and probably contributes to selection for smaller hydrophobic or aromatic substrates [70].

#### GHRB

This subfamily is characterized by the broadest substrate selectivity and highest diversity in function among 2HADHs. Members of the GHRB clade bind a large variety of putative physiological substrates, as diverse as glyoxylate, hydroxypyruvate, phosphonate, D-mandalate, 2-keto-D-gluconate, phenylpyruvate, and 3,4-dihydroxyphenylpyruvate (Table 1). At the same time, they exhibit a high level of promiscuity, i.e., they accept various secondary substrates, which occasionally appear as the most efficient substrates for the most similar homologs. In terms of *k*_cat_/*K*_M_ values, they are less active than LDHDs, with maximal values of over 10^5^ M^− 1^ s^− 1^, and although they have similar substrate profiles, GHRBs usually do not accept pyruvate. Enzymes falling into this subfamily typically possess better affinity for NADPH than for NADH (e.g., GRHPR_HUMAN, HPPR_PLESU, and GHRB_ECOLI), but individual proteins are shown to work more efficiently with NADH (e.g., HPR1_ARATH).

This heterogeneous subfamily spans enzymes from all kingdoms of life (Fig. 5). Among its representatives are yeast mandalate dehydrogenase [24], human and archaeal glyoxylate/hydroxypyruvate reductases [4, 72], bacterial phosphonate dehydrogenases [23], plant and fungal hydroxyphenylpyruvate reductases [6, 73], and bacterial enzymes reducing broad ranges of substrates [5, 69].

As hydroxypyruvate and glyoxylate constitute important compounds in various metabolic pathways, GHRB members play crucial roles in many biological processes. Mammalian glyoxylate reductase has a potentially protective role by metabolizing glyoxylate to the less reactive glycolate [4]. Hydroxyphenylpyruvate reductase from *Coleus blumei* (HPPR_PLESU) is involved in rosmarinic acid biosynthesis [73], while hydroxypyruvate reductases from *A. thaliana* (HPR1_ARATH, HPR2_ARATH, and HPR3_ARATH) are involved in photorespiratory metabolism [74]. In methylotrophic organisms, hydroxypyruvate reductase (DHGY_HYPME) plays a central role in carbon assimilation, converting hydroxypyruvate to glycerate as an essential step in the serine cycle [75]. 2-keto-D-gluconate dehydrogenase from *Gluconobacter oxydans* (2KGR_GLUOX) is responsible for the utilization of the compound as a carbon source [76].

The recent age of the duplications and evidence of a horizontal gene transfer in the recent history of the GHRB subfamily suggest that the function and enzymatic behavior of its members could be extrapolated to a limited extent. Surprisingly, some genomes encode as many as six GHRB paralogs (Fig. 5). In the N_2_-fixing ɑ-proteobacterium *Sinorhizobium* sp. NGR234, a majority of the GHRB homologs are expressed at relatively low levels (i.e., less than 100 reads per kilobase per million mapped reads, RPKM) [77]. As shown for PprA from *Wickerhamia fluorescens* TK1, their transcription could adapt to some specific metabolic conditions [6].

The enzymatic diversity of GHRB is reflected by the active site environments in the known crystal structures. Presence of the Gly-Ser motif within the βG-α5 loop is correlated with the highest activity with hydroxypyruvate (e.g., *Pyrococcus horikoshii* GYAR_PYRHO, *H. sapiens* GRHPR_HUMAN, *Plectranthus scutellarioides* HPPR_PLESU, and *S. meliloti* Q92LZ4_RHIME). The serine side chain (e.g, Ser296 in PDB ID: 2gcg and Ser280 in PDB ID: 5v7n, Additional file 8: Figure S4) is thought to be responsible for discrimination for hydroxypyruvate due to the formation of a hydrogen bond with its hydroxyl group [70]. Another highly conserved motif – Arg-X-X-Met – is located within the ɑ5 loop in most GHRB members. Probably the large side chains of the Arg and Met residues prevent the binding of substrates containing larger C2 substituents that extend the C1-C2 plane (e.g., PDB ID: 5v7n, Additional file 8: Figure S4). In *Rhodotorula graminis* Q7LLW9_RHOGR, the motif corresponds to Phe-His-Glu-Phe and correlates with high activity of the enzyme towards D-mandalate. Another important residue contributes from the other subunit of the dimer (Trp141 in PDB ID: 2gcg, Trp134 in PDB ID: 4e5k, Additional file 8: Figure S4). This large aromatic residue potentially precludes the binding of larger substrates: its absence in *S. meliloti* Q92LZ4_RHIME coincides with activity for larger substrates, such as 2-keto-D-gluconate [70]. Variable residues within the β3-α3 loop may be also involved in distinguishing physicochemical properties of the substrate. Large hydrophobic side chains, such as Leu59 in GRHPR_HUMAN or Leu70 in HPR1_ARATH, might prevent binding of substrates with large substituents, whereas small hydrophilic residues, such as Ser50 in Q92LZ4_RHIME, might promote selection towards large hydrophilic substrates.

#### PTXD

In a majority of the reconstructed phylogenetic trees, the GHRB subfamily contains a small clade, PTXD, containing (among others) phosphonate dehydrogenase from *Pseudomonas stutzeri* (PTXD_PSEST). The enzyme catalyzes the oxidation of phosphite to phosphate coupled to the stoichiometric reduction of NAD^+^ to NADH; besides, it was shown to reduce hydroxypyruvate at a low level [23]. None of other tested compounds were reduced by the enzyme; however, it has not been tested against glyoxylate and phenylpyruvate, which are common substrates for GHRB members.

Several structures of *P. stutzeri* PtxD variants with improved thermostability and catalytic efficiency have been solved (Additional file 8: Figure S4, PDB ID: 4e5k) [78, 79]. It was suggested that highly hydrophobic residues that interact with the substrate analog (Met53, Leu75, and Leu100 in PTXD_PSEST) contribute to closing off the active site [79]. The tight substrate-binding pocket is shielded by Trp314 provided by the other subunit of the dimer. However, mutagenesis studies indicate that the tryptophan is not important for catalysis [80]. Another residue from the active site, Arg301 located within helix ɑ5, is thought to contribute to electrostatic interactions with negatively charged substrates [80]. It is fully conserved in PTXD homologs, but not in other 2HADH enzymes. Interestingly, the R301K mutant displayed a slightly higher *k*_cat_ than the parent PTXD, and a more modest increase in *K*_M_ for phosphite [80]. Although three other residues—Trp314 (mentioned above), Tyr139, and Ser295—are specific for PTXD orthologs, site-directed mutagenesis proved them not important for the catalysis [80].

#### GHRC

In addition to GHRA, GHRB, and DDH, another clade of bacterial and archaeal proteins, here termed GHRC, emerged to include a glyoxylate/hydroxypyruvate reductase. The only biochemically characterized member of the subfamily—an enzyme from a methylotroph *Methylobacterium extorquens* (DHGY_METEA)—was shown to reduce hydroxypyruvate and glyoxylate, and to catalyze the reverse reaction with glycerate [19]. It was proposed to play a central role in the assimilation of carbon in methylotrophs, as it converts hydroxypyruvate to glycerate (a key step in the serine cycle) [19]. The enzyme was shown to utilize both NADH and NADPH as a cofactor. However, it is not known to what extent the characteristics of this enzyme apply to other members of this subfamily. The region responsible for cofactor specificity (i.e., the βB-αD loop) does not contain the characteristic aspartate residue that defines the preference for NADH, suggesting that NADPH may be the preferred cofactor (Fig. 3).

A crystal structure of a GHRC representative from *Desulfovibrio vulgaris* has been solved (PDB ID: 5tx7). Although the structure is in apo form, arrangement of the domains suggests that it adopts a closed conformation. The tight substrate pocket is lined with two tryptophan residues (Trp135 and Trp288), absolutely conserved in the GHRC subfamily, and a lysine residue (Lys52, Additional file 8: Figure S4). The lysine is located within the “Asn-Lys” motif at the β3-α3 loop and is present in almost all GHRC sequences. The large polar environment created by large aromatic residues is rarely seen in other subfamilies (Additional file 8: Figure S4), and may be used for other small and hydrophilic substrates not tested in the previous biochemical assay.

#### LDHD

Enzymes from this subfamily can be found in bacteria and some lower eukaryotes, such as protists, fungi and green algae. Bacterial proteins initially annotated as D-lactate dehydrogenases (LDHD, or D-LDH) act at the last step of glycolysis in anaerobic conditions, by catalyzing the reduction of pyruvate to D-lactate, allowing regeneration of NAD^+^ from NADH [81]. These enzymes may also play a role in other processes, as demonstrated for VanH, which is responsible for vancomycin resistance in *Enterococcus faecium* [82]. The only eukaryotic D-LDH studied so far, the *Chlamydomonas reinhardtii* enzyme, was shown to reduce pyruvate in fermentation pathways in chloroplasts [83]. According to the proposed role of NAD^+^ regeneration, this subfamily has the highly conserved characteristic aspartate residue that defines the preference towards NADH in the the βB-αD loop (Fig. 3).

According to our phylogenetic analysis, there are two subgroups within this subfamily: a Bacilli-specific clade (e.g., LDHD_LACPL) and another one comprising other bacteria (e.g., LDHD_ECOLI) and eukaryotes (B0LUZ5_CHLRE, Fig. 1). LDHD members usually exhibit the highest catalytic efficiency towards pyruvate (with *k*_cat_/*K*_M_ over 10^6^ M^− 1^ s^− 1^) and were also shown to accept other small compounds, such as glyoxylate and 2-ketobutyrate, with considerably lower efficiency. One exception is D-2-hydroxyisocaproate dehydrogenase (*R*-HicDH) from *Lactobacillus casei* (Q03CR3_LACC3, DHD2_LACPA), which clusters closely with typical Bacilli D-lactate dehydrogenases. In a systematic screening, *R*-HicDH was shown to reduce a broad range of substrates, including straight and branched aliphatic 2-keto acids, with phenylpyruvate and 2-ketoisocaproate with the highest *k*_cat_/*K*_M_ and *K*_M_, respectively [84]. The *k*_cat_/*K*_M_ value was three orders of magnitude lower for pyruvate. *R*-HicDH slowly catalyzes reactions with medium-size carboxylates, which, unusually, do not follow conventional Michaelis-Menten kinetics, possibly due to weak substrate binding [84]. In addition, *E. coli* LDHD was shown to be inhibited in situ by the substrate in high concentrations [85].

Analyses of crystal structures describe the architectures used by LDHDs to control the size and electrostatic character of the substrate-binding site [39]. Attention was especially brought to residues from loops β3-α3, β5-αA and βG-α5 [39]. The residues at β3-α3 play a steric role in substrate selectivity: hydrophobic and aromatic phenylalanine (e.g., Phe51 in PDB ID: 3wx0, LDHD_ECOLI) is thought to prevent binding of substrates larger than pyruvate, whereas smaller leucine (e.g., Leu51 in PDB ID: 1dxy, DHD2_LACPA) and glycine (Gly54 in VANH_ENTFC) contribute towards the broader substrate specificity of LDHDs [28, 29]. The amino acids at the β5-αA and βG-α5 loops appear to be conserved within the LDHD subfamily: tyrosine and phenylalanine/tyrosine, respectively, presumably restrict the space for C2 substituents [28]. Interestingly, Arg9 from the β1-α1 loop in *R*-HicDH from *Lactobacillus paracasei* (PDB ID: 1dxy) was proposed to be responsible for the non-Michaelis-Menten kinetics observed for this enzyme. Because of its proximity to the active site, Arg9 may compete with the arginine of the catalytic triad for the substrate and lead to non-productive substrate binding (Additional file 8: Figure S4) [28]. However, this hypothesis awaits confirmation by site-directed mutagenesis studies.

#### PDXB

This small subfamily includes a group of bacterial enzymes found in ɣ-proteobacteria and Bacteroidetes, including biochemically studied PdxB from *E. coli* (PDXB_ECOLI). PdxB oxidizes 4-phospho-D-erythronate to 2-keto-3-hydroxy-4-phosphobutanoate and uses various 2-keto acids as cosubstrates, utilizing NAD^+^ as a cofactor [27, 66]. The reaction is the second step in the biosynthesis of pyridoxal phosphate — the active form of vitamin B6 [66]. The PDXB subfamily has a highly conserved characteristic aspartate residue that defines the preference towards NADH in the βB-αD loop (Fig. 3). Uniquely for 2HADHs, PDXB family proteins have two consecutive proline residues within the loop, which are spatially conserved in all the crystal structures of PDXB proteins. Another unique feature of PDXB is presence of a C-terminal dimerization domain (Additional file 7: Figure S3).

PDXB contains three members (from *Pseudomonas aeruginosa*, *Salmonella typhimurium*, and *Vibrio cholerae*) with crystal structures of the holoenzymes (PDB IDs: 2o4c, 3oet, and 5dt9 respectively). The *P. aeruginosa* PdxB structure has been solved with a substrate analog (tartaric acid) bound in the active site. Based on the structure, it was proposed that two conserved arginines and a tyrosine residue anchor the phosphate moiety of the native substrate via charge compensation and hydrogen bonds [34]. Arg44 is located within the Arg-Ser motif at the β3-α3 loop, whereas Arg346 is located in the dimerization domain unique to PDXB (PDB ID: 2o4c, Additional file 8: Figure S4). Thus, the dimerization domain likely also plays a major role in substrate recognition. The absolutely conserved residue Tyr258 is located within the βG-α5 loop at the junction between the cofactor-binding and substrate-binding domains and presumably contributes to precise positioning of the phosphate group within the active site. Notably, the proposed mode of binding vastly differs from the consensus model presented on Fig. 4.

#### Sera

3-phospho-D-glycerate dehydrogenases (PGDH, or SERA) constitute the most widespread subfamily within 2HADHs, present in almost all living organisms. They are involved in the first step of the phosphorylated pathway of L-serine biosynthesis from 3-phosphoglycerate, an intermediate of glycolysis [86]. They reversibly oxidize D-3-phosphoglycerate to hydroxypyruvic acid phosphate utilizing NAD^+^ as a cofactor [86], with the respective aspartate residue defining the specificity for NAD(H) is highly conserved within the subfamily (βB-αD loop, Fig. 3). According to the published biochemical studies, SERA members rarely catalyze other reactions.

SerA homologs were previously divided into four distinct groups — referred to as types I, II, IIIK, and IIIH [86, 87]. Although the division was based mainly on the presence of additional regulatory domains, it is also reflected by the topology of the phylogenetic tree, computed based on the alignment of the cofactor-binding and substrate-binding domains (Fig. 1).

Type I enzymes are represented by the human, *M. tuberculosis*, and *A. thaliana* proteins. They act as tetramers and share a conserved domain architecture, where substrate-binding and cofactor-binding domains are followed by two regulatory domains—an allosteric substrate-binding (“ASB”) domain and a regulatory motif recurring in many enzymes, termed aspartate kinase-chorismate mutase-TyrA (“ACT”) domain. The ACT region binds amino acids (in this case, L-serine) and functions in feedback inhibition of amino acid synthesis pathways [88]. As shown for *M. tuberculosis* PGDH, the second layer of regulation is provided by the ASB domain. It appears to modulate sensitivity to L-serine by phosphate and polyphosphate, which triggers a conversion between oligomers with different serine-sensitive states [89].

Type II PGDHs, comprising *E. coli* and yeast enzymes, contain only an additional ACT domain and also act as tetramers. As with type I enzymes, *E. coli* SerA is strongly inhibited by L-serine, which binds to the ACT domain and allosterically regulates the velocity of the catalyzed reaction [90]. It shows an exceptionally high affinity to NADH, estimated as 50 nM [91]. Unlike *M. tuberculosis* and rat SerA, the *E. coli* enzyme can also utilize α-ketoglutarate as a substrate, yet with considerably lower affinity than 3-phosphoglycerate [87, 92].

Types IIIK and IIIH indicate type III dehydrogenases, which do not contain additional regulatory domains, with either lysine or histidine in the active site, respectively. Type III enzymes function as dimers, as opposed to type I and II, which are active as tetramers [86]. Type IIIK proteins are present in Bacteroidetes and protists, including *Entamoeba histolytica* [93]. According to crystal structures (PDB ID: 4njo) and mutagenesis studies [47], the active site is formed by arginine and lysine residues (instead of the typical Arg/His/Glu triad). Lysine is thought to be an acid-base catalyst in the reaction, taking over the role of the catalytic histidine-glutamine pair. The type IIIH enzymes are present in bacteria and archaea, including proteins with a determined 3D structure from *P. horikoshii* (PDB ID: 1wwk) and *Sulfolobus tokodaii* (PDB ID: 2ekl).

In the reconstructed ML tree, 3-sulfolactate dehydrogenase SlcC from *Chromohalobacter salexigens* (SLCC_CHRSD) is grouped within the SERA clade. However, this tree topology is not consistent among trees computed using alternative methodologies; plausibly, SLCC_CHRSD could also be placed as a sister clade to SERA (Additional file 3: Data file S1). 3-sulfolactate is structurally similar to 3-phosphoglycerate, yet SlcC is involved in another pathway using 3-sulfolactate as a carbon source and does not accept 3-phosphoglycerate as a substrate [94].

Despite the relative sequence variability in the SERA subfamily, the available crystal structures show similar solutions for recognition of the negatively charged substrate, which is based on the presence of at least two conserved positively charged residues positioning the phosphate moiety (Additional file 8: Figure S4). In particular, the Arg-Ser motif located within the β3-α3 loop, also present in the PDXB subfamily (Fig. 3), accommodates a SERA-invariant arginine that directly binds phosphate in the crystal structures. The arginine is usually stabilized by a conserved glutamine residue located in the α5 helix. The structural regions providing additional Arg/Lys residues depend on the SERA subtypes: In types I and IIIH/K, an arginine contacting the substrate is located in the β4-α4 loop, whereas in all types except for IIIK, an extra Arg/Lys residue enters the active site from the other subunit within the αB′-βA′ loop (Additional file 8: Figure S4).

#### New uncharacterized subfamilies

The 13 newly defined subfamilies that were not studied biochemically (X1-X13) constitute small clades, each comprising closely related species from bacteria, archaea, and plants (Fig. 1, Additional file 2: Figure S1). According to the sequence alignment, almost all new clades retain the conserved residues of the active site, thereby suggesting that these proteins probably act as active dehydrogenases or reductases (Additional file 7: Figure S3). Only the X13 subfamily lacks the conserved glycine residue (Gly73 in the β4-α4 loop) that provides the amide atom to position the substrate relative to the cofactor, and ~ 50% of members of the X4 subfamily have the histidine residue of the catalytic triad replaced with tryptophan, which is accompanied by a loss of the catalytic glutamate.

Seven of the newly defined subfamilies include representatives with a determined 3D structure (Additional file 2: Figure S1). Among these structures, one was solved with a cofactor (PDB ID: 1qp8 from X4), another with a cofactor and a reaction product (PDB ID: 3 kb6 from X9, Additional file 8: Figure S4); the rest of the structures do not have function-relevant ligands (Additional file 6: Table S2). Below, we provide a short description of the two subfamilies that have at least one structure in complex with a cofactor.

The X4 subfamily consists of archaeal enzymes from Crenarchaeota and Euryarchaeota. The only structure in complex with a cofactor from this subfamily (PDB ID: 1qp8) corresponds to a protein from *Pyrobaculum aerophilum* PAE1175; it is annotated as a putative formate dehydrogenase in the PDB and as a 2-hydroxyacid dehydrogenase in UniProt. Notably, it lacks the catalytic His and Glu/Gln, instead containing Trp and Val in these positions, respectively. These substitutions are unique features of about 50% of the members of this subfamily (see section: Residues of the active site). Trp is conserved among PAE1175 homologs from *Crenarchaeota*, leading to questions regarding the molecular function of these enzymes. The substrate-binding domain of PAE1175 possesses a deteriorated version of the Rossmann fold, with a short hydrogen-bonded turn instead of α2 and a short β-bridge instead of β3. Importantly, it lacks features characteristic of FDH subfamily members, such as a long β3-α3 loop with the PF[HW]P sequence motif (Fig. 3) and Ile/Val within the β4-α4 loop (Additional file 8: Figure S4). Therefore, as was concluded from an earlier analysis of its crystal structure, its role as a formate dehydrogenase seems highly far-fetched [45]. However, since formate dehydrogenation is the only reaction catalyzed by 2HADH that does not involve proton transfer, it still may be the most feasible function of the Trp-substituted enzymes from the X4 subfamily. Interestingly, X4 members from Euryarchaeota, such as Ta0858 from *Thermoplasma acidophilum* with solved structure (PDB ID: 3gvx) possess the canonical Arg-Glu-His catalytic triad. According to the pattern of sequence conservation, most members of the subfamily likely display a preference for NADP(H) over NAD (H), as the topology of the βB-αD loop is highly similar to that of GHRA (Fig. 3).

The only structure of an X9 subfamily protein with a co-factor (PDB ID: 3 kb6) corresponds to aq_727 from *Aquifex aeolicus* and is annotated as D-lactate dehydrogenase in both PDB and UniProt. X9 is a sister group to LDHD (Fig. 1), although representatives of the two subfamilies display relatively low sequence identity (< 40%). 3 kb6 was determined in complex with NAD(H) and lactic acid (the authors suggested that it could also be pyruvate, but the electron density clearly suggests that the C2 atom of this molecule is sp^3^ hybridized) [41], which interacts with the residues in a similar fashion as those seen in LDHD structures (e.g., PDB ID: 4cuk, Additional file 8: Figure S4). Therefore, despite a lack of biochemical evidence, X9 may be regarded as a new subgroup of D-lactate dehydrogenases.

### 2HADHs knowledgebase

To simplify navigation over the 2HADHs classification, we have created a software tool that converts spreadsheets containing results of our analyses into a web-based knowledgebase. The 2HADHs knowledgebase consists of three elements: an explorable phylogenetic tree of the family, an interactive table with annotations of the selected enzymes, and a BLAST search tool. Main clades on the phylogenetic tree are hyperlinked to the table with annotations of the family representatives. Protein annotations include a list of kinetically characterized substrates, highest efficiency substrate, PDB identifiers, structure ligands, and publication references for structural and kinetics studies. The protein table content can be sorted by any column and filtered by source organism kingdom, availability of kinetics, or structural studies. All proteins that have the corresponding publication or an experimentally-determined structure are hyperlinked to PubMed and the Protein Data Bank, respectively. In addition, we have generated Molstack [95] visualizations of active sites for all proteins having a cofactor and a ligand bound in this region. Molstack interactive visualizations give an instant insight into the quality of macromolecule model and a corresponding electron density map. The knowledgebase allows to classify an uncharacterized sequence and find its closest studied homologs by using a built-in BLAST tool. Its content is generated automatically from annotation spreadsheets, what makes it is easy to maintain the information up-to-date. The 2HADHs knowledgebase is publicly available at http://2hadh.bioreproducibility.org/.

## Discussion

Motivated by recent advances in genetic engineering and new societal needs, the use of enzymes as catalysts to synthesize compounds and materials is rapidly expanding. It is apparent that enzyme promiscuity offers great opportunities in the design and development of new catalytic functions in the scaffold of stable enzymes [96]. Exploiting enzyme substrate promiscuity might lead to improvements in existing catalysts and provide novel synthesis pathways that are currently not available. The D-2-hydroxyacid dehydrogenases (2HADHs) may be considered as another protein family in which substrate promiscuity and moderate-to-high efficiency are a rule rather than an exception. This feature has already been used in systems for highly stereoselective production of selected chiral α-hydroxy carboxylic acids [11, 12]. The comprehensive understanding of sequence-structure-function information provides a foundation for future biotechnological developments.

A refined evolutionary analysis and classification of the 2HADH family to large extent uphold the previous observation that most enzymes remain to cluster according to their preferential substrates. Moreover, the topology of the refined tree suggests that, besides previously determined six subfamilies (SERA, FDH, CTBP, PDXB, LDHD, and GHRB), three others (GHRA, GHRC, and DDH) with at least one biochemically characterized member could be established. Moreover, we define 13 other small subfamilies of dehydrogenases that have not been characterized biochemically. We suggest that studying representatives of these subfamilies can greatly help annotation of metabolic pathways of multiple organisms, as well as may lead to discovery of enzymes with novel biotechnological applications.

To facilitate application of the collected information, we provide a publicly available 2HADH enzymes knowledge portal, which allows to classify uncharacterized members and gives insights into the evolutionary history of substrate specificity of these heterogeneous enzymes. Although several databases have been developed to store kinetic parameters of enzymes obtained in steady states (e.g., BRENDA [97], MetaCyc [98], or UniProt [99]), for a majority of the protein families, the data are usually sparse and encompass only subsets of possible substrates. Therefore, to limit the bias and grasp the level to which the functional annotations can be extra- or interpolated, the data should be interpreted in the evolutionary context of the whole protein family. The large number of paralogs, recent duplications and horizontal transfers make function prediction within the 2HADH family particularly troublesome. To facilitate usage of the collected information, the 2HADH portal is equipped with an interface to search for close homologs within the representative enzymes and an interactive annotation data table. The portal is designed to be easily maintained and adaptable to the presentation of similar analysis of other protein families. The 2HADH knowledgebase is available at: http://2hadh.bioreproducibility.org/.

## Conclusions

We present a revised classification of the family that comprises 22 subfamilies, including 13 new subfamilies not studied biochemically. For the first time, all available enzymatic and structural features of the subfamilies were collected and analyzed in a systematic way, expanding our understanding of the features contributing to their core function of D-2-hydroxyacid dehydrogenation, as well as to their functional diversity exhibited by substrate specificities. Our family-wide sequence and structural comparison proved general importance of several active site residues that were not previously discussed in the literature (e.g., Val72, Gly73, Asn227, Gly229, and Gly231, with numbering referring to Q92LZ4_RHIME), extending our understanding of its catalytic machinery. Systematic analyses of active site environments provided key insights into the residues important (or unimportant) for substrate selectivity. In addition, these analyses have left intriguing uncertainties regarding the role of dimerization and dynamics of the secondary structure elements or entire domains, as well as the molecular mechanism for different substrate ambiguity. To facilitate usage of the collected biochemical, structural and evolutionary information, we provide a dedicated web portal allowing to classify new sequences and to generate functional hypotheses for further studies on these largely uncharacterized enzymes.

## Methods

### Phylogenetic analysis

To reliably classify the 2HADH family into evolutionary subfamilies, we constructed a phylogenetic tree using available sequence and structure information. Phylogenetic tree building relies primarily on a multiple sequence alignment (MSA) of sequences of interest. Standard automatic algorithms for building MSAs do not work well for sequences with low sequence identity, i.e., below 20–25% [100], but could be considerably improved by incorporating structural information [101]. For example, sequence identity between *A. thaliana* FDH and *H. sapiens* CTBP1 is 18%, as obtained from a global Needleman-Wunsch alignment. Given that the existing 2HADH classification relies on an MSA generated automatically with ClustalW [5, 6], we decided to improve it by using a high-quality, structure-based MSA.

First, literature searches were carried out to identify and select biochemically confirmed D-2-hydroxyacid dehydrogenases. The biochemically studied proteins were used as a “confidently annotated” reference set for the 2HADH family. Their amino acid sequences were downloaded from UniProt, trimmed to the cofactor-binding and substrate-binding domains, and used as queries for BLAST [102] against the PDB (expectation value < 10^− 5^, September 2016) to search for closely homologous enzymes with solved structures. 30 selected PDB representatives were used to create a high-quality, structure-based sequence alignment with PROMALS3D [101] using the default parameters. The “seed” structures were chosen to diversely represent the 2HADH sequence space (median identity between sequences was 23%).

To extend the sequence set, the reference 2HADH enzymes with biochemical or structural information was then used as queries in BLAST searches against 111 representative proteomes downloaded from the KEGG GENOME (Additional file 9: Data file S2). Hits with *E*-value < 10^− 3^ to at least one reference sequence and with coverage at least 90% of the query sequence were extracted and aligned with MAFFT 7.123 [100] (“mafft-linsi --add”) to the structure-based alignment of the representative structures. The alignment of 462 sequences was manually checked in SeaView 4.5.4 [103], and trimmed with trimAl to remove columns with gaps in at least 80% sequences (“trimal -gt 0.2”) [104].

Based on the resulting MSA of 462 sequences, we built phylogenetic trees using several approaches. Phylogenetic inference was carried out using neighbor-joining (NJ) and maximum-likelihood (ML) methods. The NJ tree was calculated with BioNJ [105] (Poisson distance, 100 bootstrap replicas, and JTT model). The ML trees were computed with FastTree 2.1.7 [106] (WAG+CAT evolutionary model, discrete gamma model with 20 rate categories and Shimodaira-Hasegawa test for estimation of local support values) and RAxML 8.2.7 [107] (100 bootstrap replicas, WAG evolutionary model, and estimated gamma distribution parameter: “-T 100 -f a -m PROTGAMMAWAG -p 12345 -x 12345 -# 100”). The obtained trees were visualized with Archaeopteryx [108].

### Structure analysis

Crystal structures deposited in the PDB were collected with BLAST via the RCSB PDB RESTful interface [109], using sequences of the functionally annotated 2HADHs as queries (with E-value threshold of 10^− 5^). The structures were then analyzed with BioPython [110] and PyMol [111]. The complete list of the analyzed structures can be found in Additional file 6: Table S2.

In the 40 structures solved with both a cofactor and a ligand analog bound in the active site, we mapped residues within 5 Å from the bound substrate (Additional file 8: Figure S4).

### Web server

The web server was created in JavaScript and Node.js run-time environment. The BLAST database of the 462 sequences was generated with “makeblastdb”. Sequence searching is carried out using “blastp” command with default parameters [102]. The web server is accessible at http://2hadh.bioreproducibility.org/.

## Additional files

Additional file 1: Supplementary Results. Horizontal gene transfer from bacteria to plants. (PDF 28 kb)

Additional file 2: **Figure S1.** Maximum-likelihood evolutionary tree of the 2HADH family. The branch labels correspond to UniProt accessions of proteins with studied substrate specificities (orange dots), known crystal structures (green dots), or both (red dots). The scale bar represents the number of estimated changes per position. A crystal structure of GHRC from *Desulfovibrio vulgaris* (PDB ID: 5tx7) was solved after the analysis was performed, and is not shown in the figure. (PDF 56 kb)

Additional file 3: **Data file S1.** Phylogenetic trees in Newick format. In the order of appearance in the file: 1) maximum-likelihood tree computed with FastTree, 2) maximum-likelihood tree computed with RAxML, 3) neighbor-joining tree computed with BioNJ. Node names refer to either KEGG or UniProt accessions. (TXT 59 kb)

Additional file 4: **Table S1.** Kinetic parameters for 2HADHs and prominent substrates described in the literature. (XLSX 503 kb)

Additional file 5: **Figure S2.** Kinetic parameters (a, *k*_cat_/*K*_M_; b, *K*_M_) for 2HADHs from the nine biochemically studies subfamilies. Results are illustrated as box-and-whisker plots where the thick line represents the median within the subfamilies and the box area encompasses 50% of all observations. Red dots correspond to the most efficient substrates (in terms of *k*_cat_/*K*_M_ or relative catalytic activity) for the enzymes, grey dots – to secondary substrates. (PDF 71 kb)

Additional file 6: **Table S2.** Table of 2HADHs of known crystal structure. (XLSX 495 kb)

Additional file 7: **Figure S3**. Sequence logos of all defined 2HADH subfamilies aligned with the reference sequence of *Rhizobium meliloti* GHRB (Q92LZ4). The structure-based alignment was obtained for selected structures with PROMALS3D and used as a seed alignment for other 2HADH sequences from 111 representative organisms. The sequence logos were generated with WebLogo, showing columns for which in at least one subfamily at least 90% members possess an amino acid (i.e., with at most 10% gapped positions). C-terminal fragments were cut out, except for the fragment of the PDXB dimerization domain (“PDXB-dim”). The top row denotes secondary structure elements common for the substrate-binding (i.e., α1-α5 and β1-β5) and catalytic (i.e., αA-αH and βA-βG) domains. The bottom row indicates regions potentially involved in substrate binding, with catalytic triad residues denoted by red triangles. (PDF 1582 kb)

Additional file 8: **Figure S4.** Active sites of selected 2HADH enzymes. Cofactors, substrates (or their analogs), and residues that potentially contribute to substrate specificity are shown as sticks. Carbon atoms of substrates or their analogs are shown in black. Colors of the residue labels correspond to structural regions of the proteins (see also Fig. 3). The residues of the catalytic triad are indicated with red labels. (PDF 9523 kb)

Additional file 9: **Data file S2.** List of KEGG organisms with completely sequenced genomes used for protein sequence searches. (TXT 8 kb)

## Abbreviations

2HADH: D-2-hydroxyacid dehydrogenase
CTBP: C-terminal binding proteins
DDH: broad-substrate-specificity dehydrogenases
FDH: formate dehydrogenases
GHRA: glyoxylate/hydroxypyruvate reductases A
GHRB: glyoxylate/hydroxypyruvate reductases B
GHRC: glyoxylate/hydroxypyruvate reductases C
GRHPR: human glyoxylate reductase
*K*_1/2_: the concentration of substrate that produces a half-maximal enzyme velocity under the sigmoidal model of kinetics
*k*_*cat*_: turnover number
*K*_*M*_: Michaelis constant, i.e., the concentration of substrate that produces a half-maximal enzyme velocity under the Michaelis-Menten model
LDHD: D-lactate dehydrogenases
ML: maximum-likelihood
MSA: multiple sequence alignment
NAD^+^: nicotinamide adenine dinucleotide
NADP^+^: Nicotinamide adenine dinucleotide phosphate
NJ: neighbor-joining
PDB: Protein Data Bank
PDXB: 4-phosphoerythronate dehydrogenases
SERA: 3-phosphoglycerate dehydrogenases
X1–X13: new 2HADH subfamilies
