# Correction to: Classification, substrate specificity and structural features of D-2-hydroxyacid dehydrogenases: 2HADH knowledgebase

**DOI:** 10.1186/s12888-019-2207-3

**Published:** 2019-07-16

**Authors:** Dorota Matelska, Ivan G. Shabalin, Jagoda Jabłońska, Marcin J. Domagalski, Jan Kutner, Krzysztof Ginalski, Wladek Minor

**Affiliations:** 1 Department of Molecular Physiology and Biological Physics, University of Virginia, 1340 Jefferson Park Avenue, Charlottesville, VA 22908 USA; 2 Laboratory of Bioinformatics and Systems Biology, Centre of New Technologies, University of Warsaw, Zwirki i Wigury 93, 02-089 Warsaw, Poland; 3 Center for Structural Genomics of Infectious Diseases (CSGID), Charlottesville, VA 22908 USA; 4 Department of Chemistry, Laboratory for Structural and Biochemical Research, Biological and Chemical Research Centre, University of Warsaw, Zwirki i Wigury 101, 02-089 Warsaw, Poland; 5 Department of Chemistry, University of Warsaw, Ludwika Pasteura 1, 02-093 Warsaw, Poland

**Correction to: BMC Evol Biol**

**https://doi.org/10.1186/s12862-018-1309-8**

Following publication of the original article [1], we have been notified that some important information was omitted by the authors from the Competing interests section. The declaration should read as below.

One of the authors (WM) notes that he has also been involved in the development of state-of-the-art software, data management and mining tools; some of them were commercialized by HKL Research.

None of commercial software was mentioning in the paper but because knowledge gained during research may be in future used in commercial software, WM has disclosed a financial interest in this research as WM is the co-founder of HKL Research and a member of the board.

The authors have no other relevant affiliations or financial involvement with any organization or entity with a financial interest in or financial conflict with the subject matter or materials discussed in the manuscript apart from those disclosed.
