# Supplementary material for: Classification, substrate specificity and structural features of D-2-hydroxyacid dehydrogenases: 2HADH knowledgebase
Source: BMC Evol Biol. 2018 Dec 22;18:199. doi: 10.1186/s12862-018-1309-8 (PMC6303947; doi:10.1186/s12862-018-1309-8)
Supplement: Supplementary file 1 — Supplementary Results. Horizontal gene transfer from bacteria to plants. (PDF 28 kb) [file 12862_2018_1309_MOESM1_ESM.pdf]

Supplementary Results

Horizontal gene transfer from bacteria to plants

The topology of the phylogenetic tree of the GHRB clade does not follow the phylogeny of the tree of life derived by analysis of ribosomal RNA and other universal genes [1]. In particular, the topology of the GHRB clade suggests a possible premise for a horizontal gene transfer (HGT) from α-proteobacteria to plants. A BLAST search using the sequence of hydroxyphenylpyruvate reductase from the plant *Coleus blumei* (HPPR_PLESU) as a query identified several closely homologous enzymes from other core angiosperms, mostly monocots and eudicots, including multiple nodulating plants. These enzymes form the homologous cluster comprising two *A. thaliana* hydroxypyruvate/glyoxylate reductases, HPR2 and HPR3, respectively. The highest scoring hits after plant proteins appeared to be α-proteobacterial proteins, with *Agrobacterium* homologs in the top (55% sequence identity), suggesting that the α-proteobacteria transferred the genes to the plants. The most similar bacterial proteins that have been studied experimentally are 2-keto-ᴅ-gluconate dehydrogenase from *Gluconobacter oxydans* (2KGR_GLUOX) and hydroxypyruvate/glyoxylate reductase from *S. meliloti* (Q92LZ4_RHIME), which also possesses activity for 2-keto-ᴅ-gluconate. In *Oryza sativa*, the potential HGT was followed by extensive recent gene duplications of the transferred gene, as suggested by high sequence similarity between its paralogs.

The postulated HGT might have occurred just after early diversifications of mesangiosperms [2], in a common ancestor of monocots and eudicots. As the closest bacterial proteins belong to α-proteobacteria symbiotic with plant roots, it might be a consequence of symbiosis with N_2-fixing α-proteobacteria, dated at around 100 million years ago [3]. Numerous cases of HGT from bacteria to eukaryotes have been demonstrated previously, although this process is assumed to be much less frequent than HGT between bacteria [4]. A case of a similar interdomain transfer was reported previously for γ-glutamylcysteine ligases [5].

Additional references

1. Hug LA, Baker BJ, Anantharaman K, Brown CT, Probst AJ, Castelle CJ, Butterfield CN, Hernsdorf AW, Amano Y, Ise K *et al*. A new view of the tree of life. Nat Microbiol. 2016, 1:16048.

2. Davies TJ, Barraclough TG, Chase MW, Soltis PS, Soltis DE, Savolainen V. Darwin's abominable mystery: Insights from a supertree of the angiosperms. Proc Natl Acad Sci U S A. 2004, 101(7):1904-1909.

3. Werner GD, Cornwell WK, Sprent JI, Kattge J, Kiers ET. A single evolutionary innovation drives the deep evolution of symbiotic N2-fixation in angiosperms. Nature Communications. 2014, 5:4087.

4. Koonin EV. Horizontal gene transfer: essentiality and evolvability in prokaryotes, and roles in evolutionary transitions. F1000Research. 2016, 5:1805.

5. Copley SD, Dhillon JK. Lateral gene transfer and parallel evolution in the history of glutathione biosynthesis genes. Genome Biology. 2002, 3(5).
