# Supplementary figures and images for: Classification, substrate specificity and structural features of D-2-hydroxyacid dehydrogenases: 2HADH knowledgebase
Source: BMC Evol Biol. 2018 Dec 22;18:199. doi: 10.1186/s12862-018-1309-8 (PMC6303947; doi:10.1186/s12862-018-1309-8)

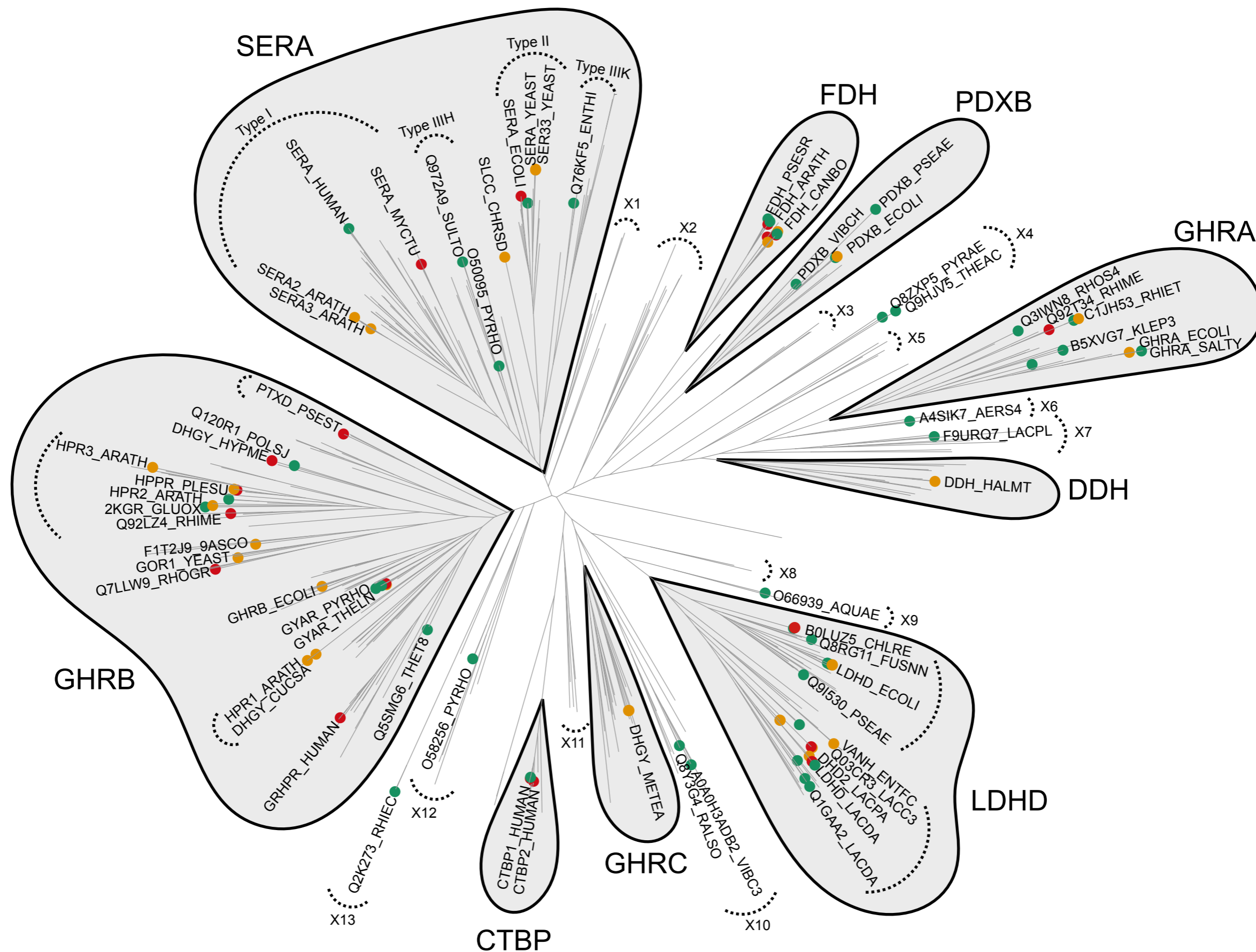

Supplement: Supplementary file 2 — Figure S1. Maximum-likelihood evolutionary tree of the 2HADH family. The branch labels correspond to UniProt accessions of proteins with studied substrate specificities (orange dots), known crystal structures (green dots), or both (red dots). The scale bar represents the number of estimated changes per position. A crystal structure of GHRC from Desulfovibrio vulgaris (PDB ID: 5tx7) was solved after the analysis was performed, and is not shown in the figure. (PDF 56 kb) [file 12862_2018_1309_MOESM2_ESM.pdf]

**A**

### Catalytic efficiencies in the subfamilies

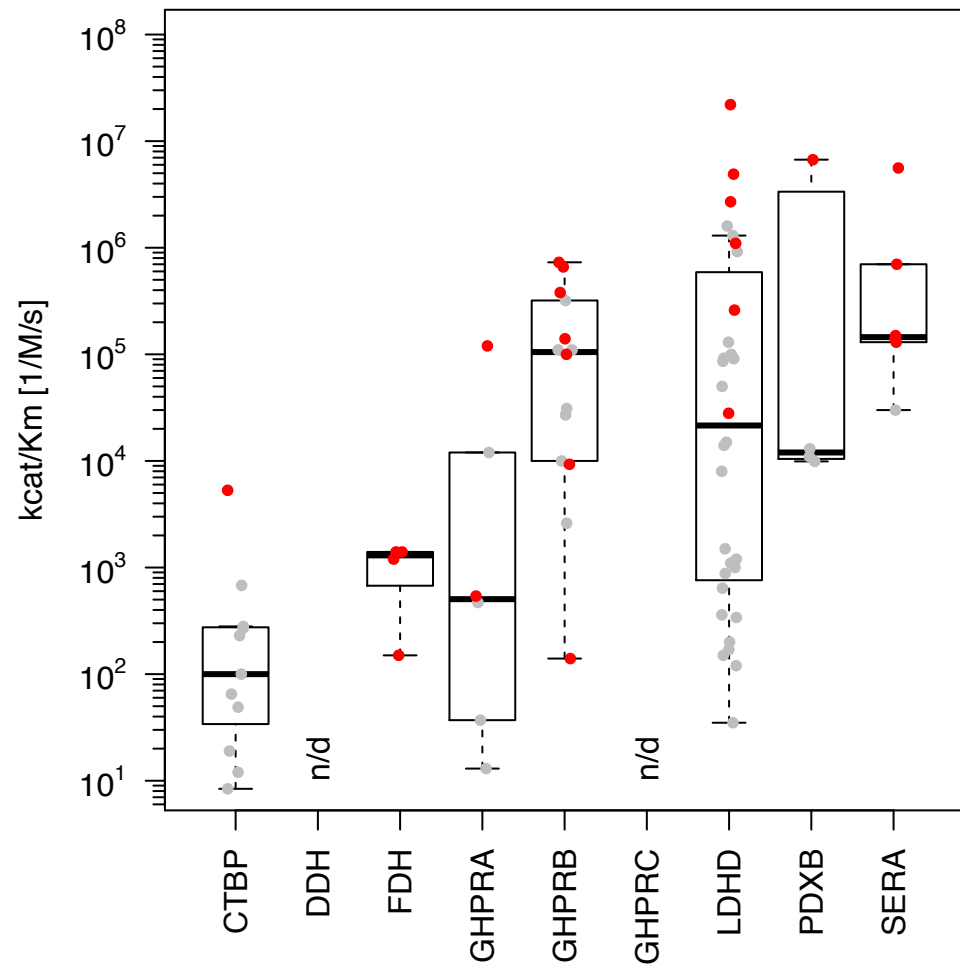**B**

### Affinities for substrates in the subfamilies

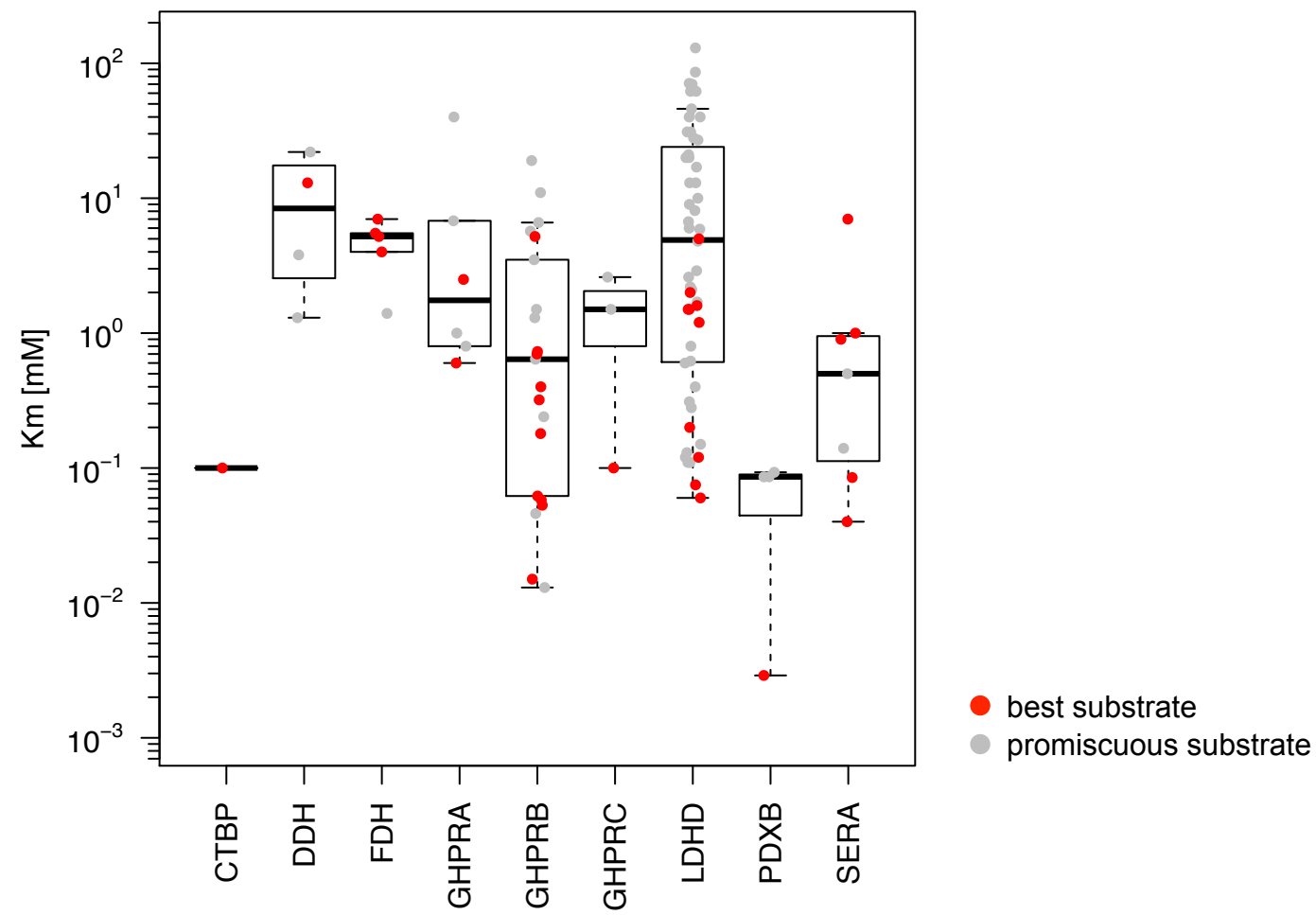

Supplement: Supplementary file 5 — Figure S2. Kinetic parameters (a, kcat/KM; b, KM) for 2HADHs from the nine biochemically studies subfamilies. Results are illustrated as box-and-whisker plots where the thick line represents the median within the subfamilies and the box area encompasses 50% of all observations. Red dots correspond to the most efficient substrates (in terms of kcat/KM or relative catalytic activity) for the enzymes, grey dots – to secondary substrates. (PDF 71 kb) [file 12862_2018_1309_MOESM5_ESM.pdf]
