# Supplementary material for: Classification, substrate specificity and structural features of D-2-hydroxyacid dehydrogenases: 2HADH knowledgebase
Source: BMC Evol Biol. 2018 Dec 22;18:199. doi: 10.1186/s12862-018-1309-8 (PMC6303947; doi:10.1186/s12862-018-1309-8)
Supplement: Supplementary file 7 — Figure S3. Sequence logos of all defined 2HADH subfamilies aligned with the reference sequence of Rhizobium meliloti GHRB (Q92LZ4). The structure-based alignment was obtained for selected structures with PROMALS3D and used as a seed alignment for other 2HADH sequences from 111 representative organisms. The sequence logos were generated with WebLogo, showing columns for which in at least one subfamily at least 90% members possess an amino acid (i.e., with at most 10% gapped positions). C-terminal fragments were cut out, except for the fragment of the PDXB dimerization domain (“PDXB-dim”). The top row denotes secondary structure elements common for the substrate-binding (i.e., α1-α5 and β1-β5) and catalytic (i.e., αA-αH and βA-βG) domains. The bottom row indicates regions potentially involved in substrate binding, with catalytic triad residues denoted by red triangles. (PDF 1582 kb) [file 12862_2018_1309_MOESM7_ESM.pdf]

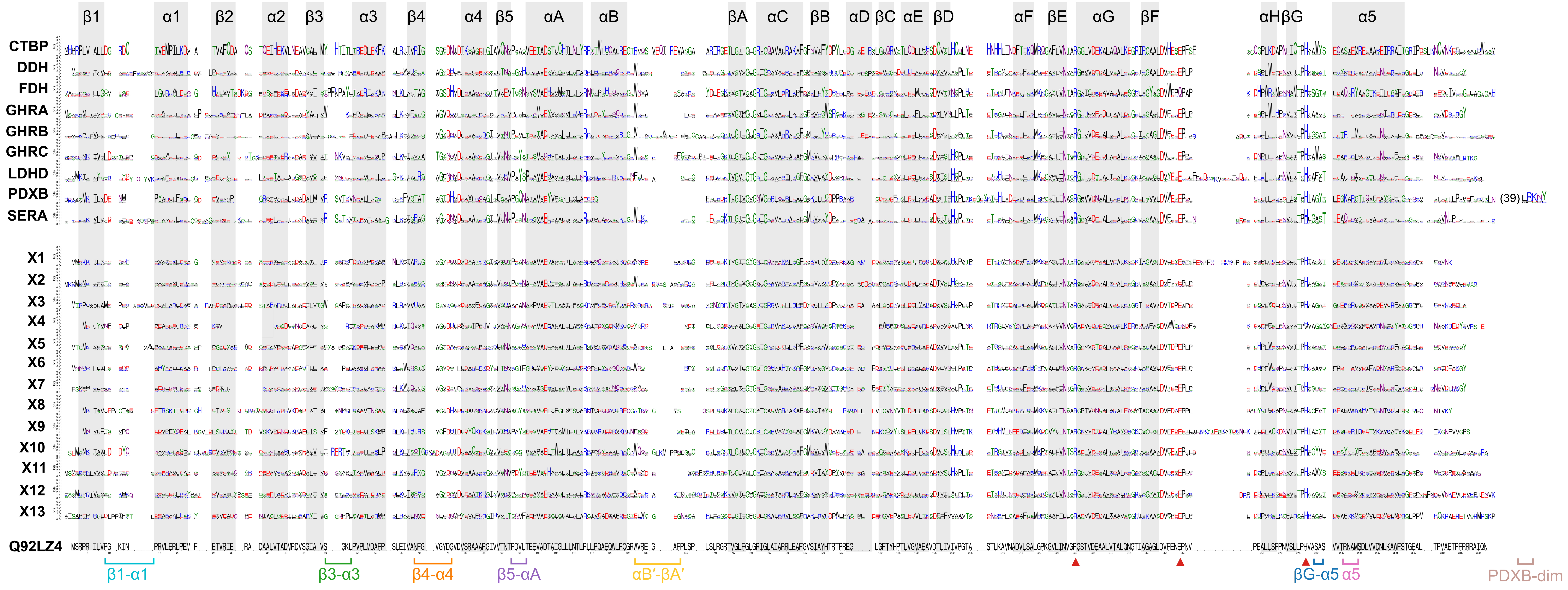
β1
α1
β2
α2
β3
α3
β4
α4
β5
αA
αB
βA
αC
βB
αD
βC
αE
βD
αF
βE
αG
βF
αHβG
α5
CTBP
DDH
FDH
GHRA
GHRB
GHRC
LDHD
PDXB
SERA
X1
X2
X3
X4
X5
X6
X7
X8
X9
X10
X11
X12
X13
Q92LZ4
(39)
β1-α1
β3-α3
β4-α4
β5-αA
αB′-βA′
βG-α5
α5
PDXB-dim
