# Supplementary material for: Classification, substrate specificity and structural features of D-2-hydroxyacid dehydrogenases: 2HADH knowledgebase
Source: BMC Evol Biol. 2018 Dec 22;18:199. doi: 10.1186/s12862-018-1309-8 (PMC6303947; doi:10.1186/s12862-018-1309-8)
Supplement: Supplementary file 8 — Figure S4. Active sites of selected 2HADH enzymes. Cofactors, substrates (or their analogs), and residues that potentially contribute to substrate specificity are shown as sticks. Carbon atoms of substrates or their analogs are shown in black. Colors of the residue labels correspond to structural regions of the proteins (see also Fig. 3). The residues of the catalytic triad are indicated with red labels. (PDF 9523 kb) [file 12862_2018_1309_MOESM8_ESM.pdf]

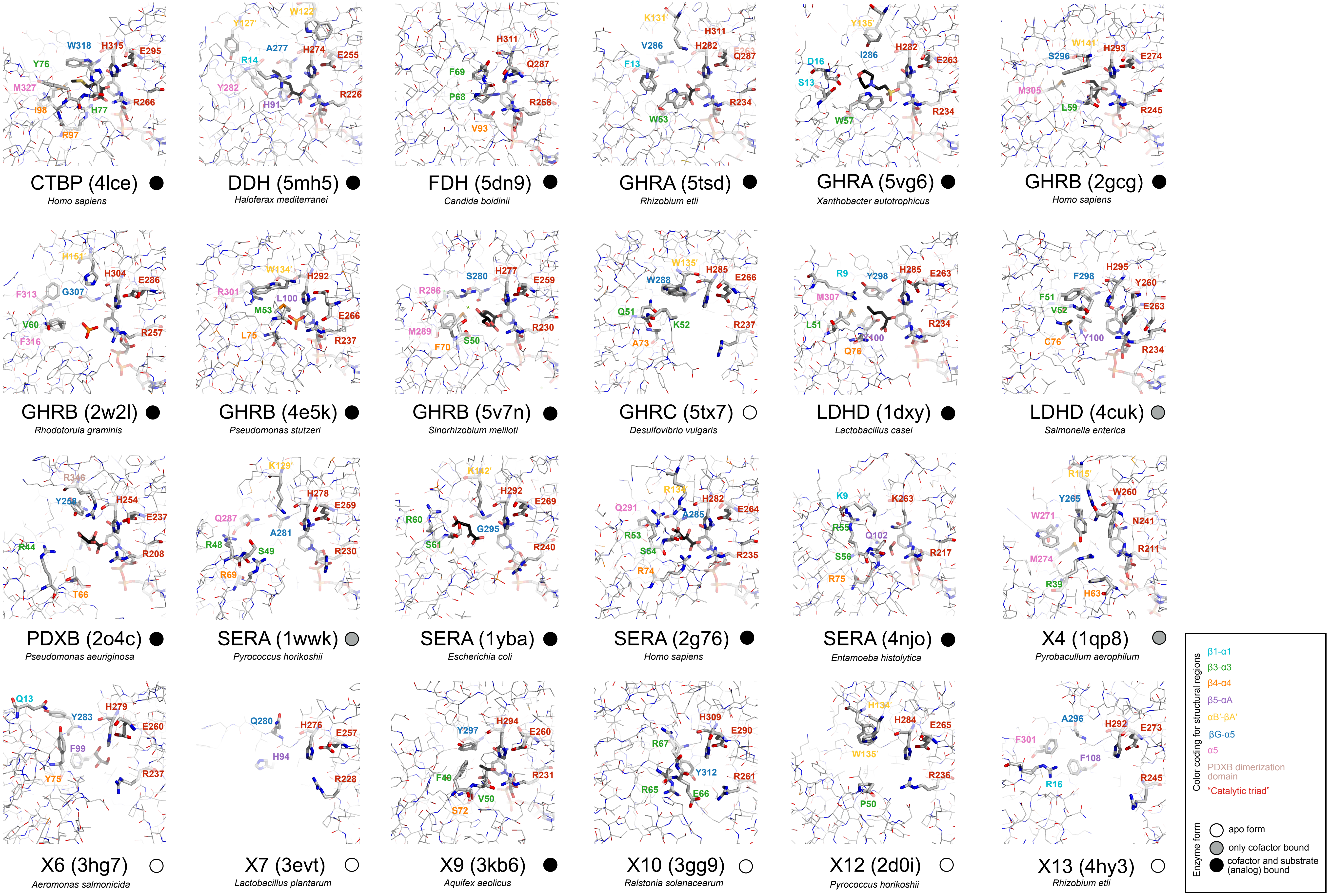

CTBP (4lce)
Homo sapiens
DDH (5mh5)
Haloferax mediterranei
FDH (5dn9)
Candida boidinii
GHRA (5tsd)
Rhizobium etli
GHRA (5vg6)
Xanthobacter autotrophicus
GHRB (2gcg)
Homo sapiens
GHRB (2w2l)
Rhodotorula graminis
GHRB (4e5k)
Pseudomonas stutzeri
GHRB (5v7n)
Sinorhizobium meliloti
GHRC (5tx7)
Desulfovibrio vulgaris
LDHD (1dxy)
Lactobacillus casei
LDHD (4cuk)
Salmonella enterica
PDXB (2o4c)
Pseudomonas aeuriginosa
SERA (1wwk)
Pyrococcus horikoshii
SERA (1yba)
Escherichia coli
SERA (2g76)
Homo sapiens
SERA (4njo)
Entamoeba histolytica
X4 (1qp8)
Pyrobacullum aerophilum
X6 (3hg7)
Aeromonas salmonicida
X7 (3evt)
Lactobacillus plantarum
X9 (3kb6)
Aquifex aeolicus
X10 (3gg9)
Ralstonia solanacearum
X12 (2d0i)
Pyrococcus horikoshii
X13 (4hy3)
Rhizobium etli
Color coding for structural regions
β1-α1
β3-α3
β4-α4
β5-αA
αB'-βA'
βG-α5
α5
PDXB dimerization domain
"Catalytic triad"
Enzyme form
apo form
only cofactor bound
cofactor and substrate (analog) bound
